# Bacterial Lipoproteins Shift Cellular Metabolism to Glycolysis in Macrophages Causing Bone Erosion

**DOI:** 10.1128/spectrum.04293-22

**Published:** 2023-05-16

**Authors:** Minh-Thu Nguyen, Zhicheng Hu, Majd Mohammad, Hannah Schöttler, Silke Niemann, Michelle Schultz, Katarzyna Barczyk-Kahlert, Tao Jin, Heiko Hayen, Mathias Herrmann

**Affiliations:** a Institute of Medical Microbiology, University Hospital Münster, Münster, Germany; b Center for Clinical Laboratories, The Affiliated Hospital of Guizhou Medical University, Guiyang, China; c Department of Rheumatology and Inflammation Research, Institute of Medicine, Sahlgrenska Academy, University of Gothenburg, Gothenburg, Sweden; d Institute of Inorganic and Analytical Chemistry, University of Münster, Münster, Germany; e Institute of Immunology, University of Münster, Münster, Germany; Texas A&M University

**Keywords:** bone marrow-derived macrophages, Pam_2_Cys, Pam_3_Cys, bacterial lipoprotein, bone erosion, cellular metabolism, lactate

## Abstract

Belonging to a group of membrane proteins, bacterial lipoproteins (LPPs) are defined by a unique lipid structure at their *N*-terminus providing the anchor in the bacterial cell membrane. In Gram-positive bacteria, LPPs play a key role in host immune activation triggered through a Toll-like receptor 2 (TLR2)-mediated action resulting in macrophage stimulation and subsequent tissue damage demonstrated in *in vivo* experimental models. Yet the physiologic links between LPP activation, cytokine release, and any underlying switches in cellular metabolism remain unclear. In this study, we demonstrate that Staphylococcus aureus Lpl1 not only triggers cytokine production but also confers a shift toward fermentative metabolism in bone marrow-derived macrophages (BMDMs). Lpl1 consists of di- and tri-acylated LPP variants; hence, the synthetic P2C and P3C, mimicking di-and tri-acylated LPPs, were employed to reveal their effect on BMDMs. Compared to P3C, P2C was found to shift the metabolism of BMDMs and the human mature monocytic MonoMac 6 (MM6) cells more profoundly toward the fermentative pathway, as indicated by lactate accumulation, glucose consumption, pH reduction, and oxygen consumption. *In vivo*, P2C caused more severe joint inflammation, bone erosion, and lactate and malate accumulation than P3C. These observed P2C effects were completely abrogated in monocyte/macrophage-depleted mice. Taken together, these findings now solidly confirm the hypothesized link between LPP exposure, a macrophage metabolic shift toward fermentation, and ensuing bone destruction.

**IMPORTANCE** Osteomyelitis caused by S. aureus is a severe infection of the bone, typically associated with severe bone function impairment, therapeutic failure, high morbidity, invalidity, and occasionally even death. The hallmark of staphylococcal osteomyelitis is the destruction of the cortical bone structures, yet the mechanisms contributing to this pathology are hitherto poorly understood. One bacterial membrane constituent found in all bacteria is bacterial lipoproteins (LPPs). Previously, we have shown that injection of purified S. aureus LPPs into wild-type mouse knee joints caused a TLR2-dependent chronic destructive arthritis but failed to elicit such effect in monocyte/macrophage-depleted mice. This observation stirred our interest in investigating the interaction of LPPs and macrophages and analyzing the underlying physiological mechanisms. This ascertainment of LPP-induced changes in the physiology of macrophages provides an important clue in the understanding of the mechanisms of bone disintegration, opening novel avenues to manage the course of S. aureus disease.

## INTRODUCTION

Bacterial lipoproteins (LPPs) have been considered a major key player of Gram-positive bacteria in the immune response through Toll-like receptor 2 (TLR2) activation (1, 2). Unlike LPPs in Gram-negative bacteria, Gram-positive bacteria produce diverse structures of LPPs, for instance, the di-acylated LPP in Listeria monocytogenes; the *N*-acetylated LPP in Bacillus subtilis, Bacillus licheniformis, Geobacillus kaustophilus, and Oceanobacillus iheyensis; the Lyso LPP in Bacillus cereus, Enterococcus faecalis, and Streptococcus pneumoniae; and the *N*-acyl-LPP (also referred to as tri-acylated LPP) in Staphylococcus epidermidis and Staphylococcus aureus (3, 4). Previous studies have demonstrated that Gram-positive bacteria modify the lipid moiety structures to evade host immune activation (4, 5). Our previous result has shown that injection of purified S. aureus LPPs into knee joints of wild-type mice caused macroscopic, chronic, and destructive arthritis as well as bone resorption but failed to elicit such an effect in a monocyte/macrophage-depleted mice (6, 7). Recently, we also demonstrated that subcutaneous injection of purified S. aureus LPPs induced skin inflammation via TLR2 with rapid infiltration of monocytes/macrophages into skin tissue in a murine model (8). Beyond the hitherto well-described, broad-spectrum functions of macrophages in the innate immune system, these novel data suggested that upon an LPP-mediated activation through their TLR2 receptors, macrophages could contribute to a more complex impact (including tissue damage) at the infection site. Hence, these observations stirred our interest in investigating the interaction of bacterial LPPs and macrophages and to analyze the underlying physiological mechanisms, including major metabolic pathway employment.

Historically, oxygen levels and nutrient supply were seen as the key drivers of metabolic pathways; however, recently, it has become apparent that immune stimuli can also cause metabolic reprogramming in cells (9). Mammalian cells use several different metabolic pathways to generate adequate levels of energy for cell growth and proliferation. Hence, different metabolic programs are utilized in different types and stages of cells, i.e., undifferentiated stem cells versus differentiated cells and proliferating versus nonproliferating cells (10). A shift of the metabolic pathways employed was also observed in host cells infected with bacteria or exposed to bacterial stimuli (10). This metabolic reprogramming during infection occurs upon eukaryotic cell exposure both by Gram-positive as well as by Gram-negative bacteria, for example, with Mycobacterium tuberculosis (11, 12), S. aureus (13), L. monocytogenes (14), Legionella pneumophila (15), Brucella abortus (16), Chlamydia trachomatis (17), and Chlamydia pneumoniae (18). In innate immune cells exposed to Gram-negative bacteria, such metabolic shift (toward the glycolytic pathway) is mediated through a lipopolysaccharide (LPS)-Toll-like receptor engagement and subsequent TLR4 activation (19). In the LPS-free Gram-positive bacteria, the signaling mechanism resulting in a metabolic shift appears also to involve TLR engagement, albeit in a manner different from employing the LPS-TLR4 axis. Indeed, M. tuberculosis induces a TLR2-dependent switch toward glycolysis in human peripheral blood mononuclear cells (PBMCs) (20).

Here, close evaluation of the bacterial LPPs with different structures of lipid moieties and their respective effect on the eukaryotic metabolic pathways engaged upon TLR2 activation is presented. Based on these data, the impact of metabolic shift by TLR2-activated macrophages causing bone erosion is proposed.

## RESULTS

### S. aureus Lpl1(+sp), but not Lpl1(−sp), induced a significantly elevated fermentation and cytokine production in BMDMs.

**FIG 1:**
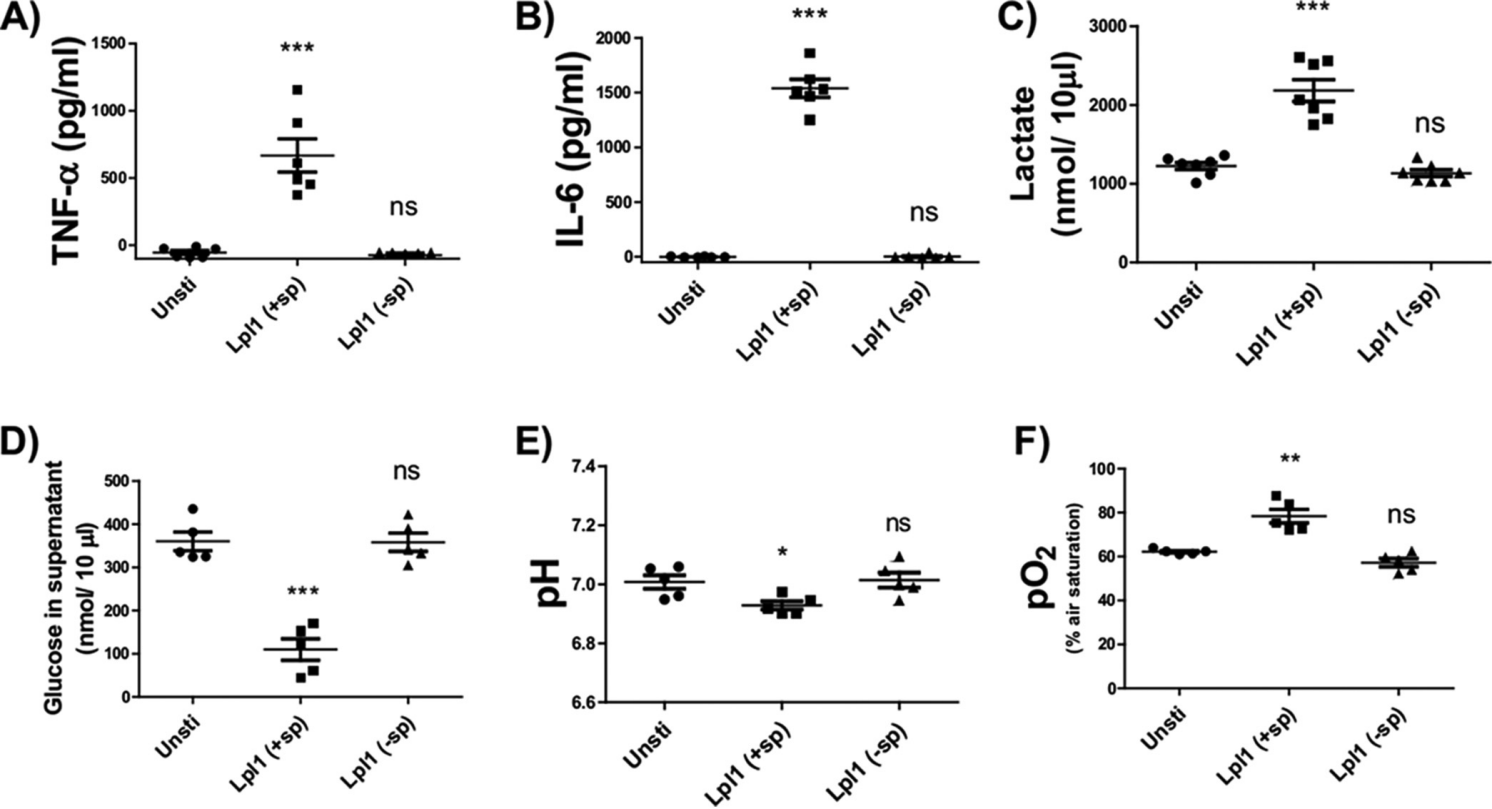
Lpl1(+sp) induced a significantly elevated fermentation and cytokine production in bone marrow-derived macrophages (BMDMs). Experimental conditions were as described in Materials and Methods, and the various assessments were performed in the supernatants of BMDM cells stimulated with 300 ng/mL of Lpl1(+sp) or Lpl1(−sp) for either 20 h (A, B) or 24 h (C to F). Control samples (unsti.) were used without adding any stimulators. The experiments were carried out 2 different times, each time conducted with 2 to 3 mice. Error bars indicate mean ± SEM. Statistical significances were calculated between the treated cells and control (unsti.) by one-way ANOVA using Tukey’s multiple-comparison test; *, *P ≤ *0.05; **, *P < *0.01; ***, *P < *0.001.

Our previous study has shown that S. aureus Lpl1, marked as Lpl1(+sp) (plus signal peptide), caused bone destruction, as well as bone resorption, in the murine model with the presence of monocytes/macrophages (6, 7); therefore, the interaction of Lpl1(+sp) with murine macrophages was investigated. In this study, the Lpl1(−sp) (minus signal peptide) lacking the lipid moiety was used to evaluate the role of the lipidated moiety versus the protein part of the lipoprotein. As expected, our previous data were confirmed, i.e., only Lpl1(+sp), but not Lpl1(−sp), induced the cytokine production of tumor necrosis factor alpha (TNF-α) and interleukin 6 (IL-6) in BMDMs (Fig. 1A and B). Interestingly, Lpl1(+sp)-treated BMDMs display significantly altered lactate concentrations in the medium compared to untreated BMDMs (Fig. 1C). Lpl1(+sp)-treated BMDMs consume more glucose, as assessed by a decreased glucose level in the medium (Fig. 1D). In line with these results, Lpl1(+sp)-treated BMDMs produced a decreased pH level compared to untreated cells. Of note, the RPMI 1640 medium was buffered with 2 g/L sodium bicarbonate to maintain the physiological pH; thus, the alteration of pH upon Lpl1(+sp) stimulation was small (below 0.2), yet the difference was significant (Fig. 1E). These results suggest that Lpl1(+sp) drives a metabolic shift in BMDM cells toward a fermentative process. To confirm this hypothesis, the percentage of oxygen in the medium (supernatant) was determined. After 24 h stimulation, the oxygen concentration in samples of BMDM cells treated with Lpl1(+sp) was determined as significantly elevated compared to untreated cells, suggesting a reduced consumption of oxygen in Lpl1(+sp)-treated cells (Fig. 1F). Taken together, only Lpl1(+sp), but not Lpl1(−sp), displays significantly altered metabolism indicators, suggesting that the lipid moiety of the lipoprotein is responsible for this modification.

### The di-acylated LPP analogue (P2C) induces a higher cytokine production than the tri-acylated LPP analogue (P3C) in BMDM and mature monocytic MonoMac 6 cells.

**FIG 2:**
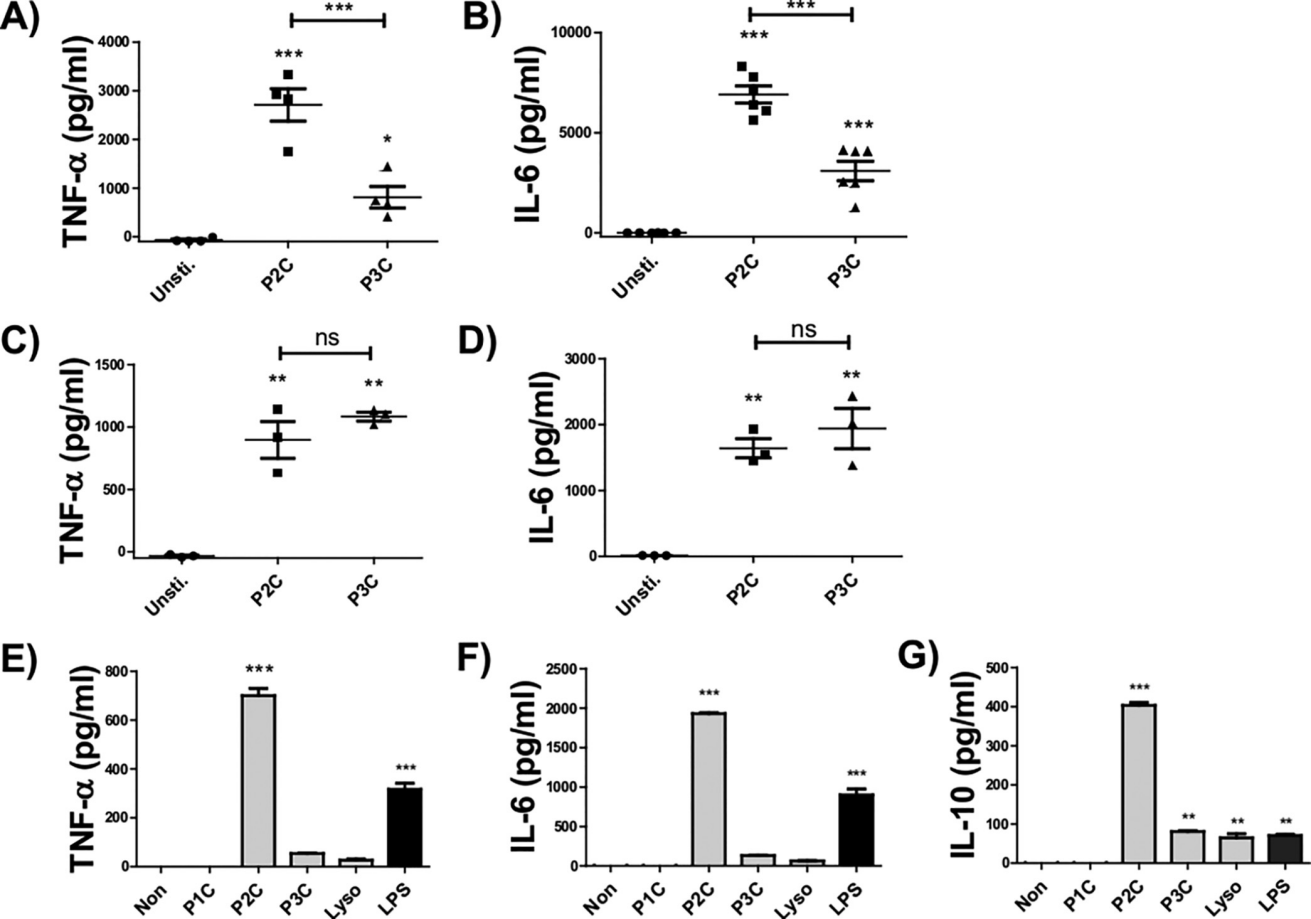
P2C induces significantly higher cytokine production than P3C in BMDM and MM6 cells. Cytokine production of BMDM (A, B) and BMM (C, D) stimulated with 100 ng/mL of indicated LPP analogues or LPS was assessed as described in Materials and Methods. Control samples (unsti.) were used without adding any stimulators. mTNF-α and mIL6 were measured by ELISA from supernatant of 20 h stimulation. The data were obtained from cells isolated from 3 to 6 mice. Error bars indicate mean ± SEM. Statistical significances were calculated between the treated cells compared to control (non) by one-way ANOVA using Tukey’s multiple-comparison test: *, *P < *0.05; **, *P < *0.01; ***, *P < *0.001. (E to G) Cytokine production of MM6 stimulated with 100 ng/mL of indicated LPP analogues or LPS was assessed as described in Materials and Methods. hTNF-α values were measured by ELISA from supernatant of 5 h stimulation, and hIL6 and hIL-10 were measured by ELISA from supernatant of 18 h stimulation. Three independent experiments were carried out in triplicate. Error bars indicate SEM. Statistical significances were calculated between the treated cells and control (unsti.) by one-way ANOVA using Tukey’s multiple-comparison test; *, *P ≤ *0.05; **, *P < *0.01; ***, *P < *0.001.

Our recent study has proven that Lpl1(+sp) is the mixture of di- and tri-acylated lipoproteins (7); therefore, the impact of lipid moiety structures on cytokine production and metabolic shift in murine monocytes/macrophages was evaluated by using synthetic peptides, i.e., P2C and P3C, as representatives for di- and tri-lipidated lipoproteins. One hundred nanograms per milliliter of either P2C and P3C could trigger the TNF and IL-6 production in BMDMs significantly; however, a biologically relevant boost of cytokine production was observed in P2C-treated BMDMs, while the treatment of BMDMs with P3C elicited a moderate increase (Fig. 2A and B). Unexpectedly, the difference of P2C versus P3C stimulation on the cytokine response was observed only in BMDMs but not in bone marrow monocytes (Fig. 2C and D), hinting toward an important role of macrophage differentiation with respect to the LPP-elicited immune response.

These results were extended and largely confirmed with the well-established and widely employed MM6 line (21–25). In addition to P2C and P3C lipopeptides, the monopalmitoylated *N*-palmitoylated peptides (Lyso) mimicking the Lyso LPPs found recently in B. cereus, E. faecalis, and S. pneumoniae (see Fig. S1 in the supplemental material) were used to stimulate with MM6 cells at a concentration of 100 ng/mL. P1C lipopeptides, which are not representing bacterial LPPs, were used as a negative control. Bacterial lipopolysaccharide (LPS), with a concentration of 100 ng/mL, was used as positive control. In line with the results obtained with murine BMDMs, MM6 cells induced with P2C displayed an exceedingly and significantly higher secretion of cytokines such as TNF-α, IL-6, and IL-10 (Fig. 2E to G). Unlike stimulation with P1C, a statistically significant, albeit very low (compared to P2C stimulation), cytokine induction was observed upon MM6 stimulation with P3C and Lyso (Fig. 2E to G). LPS induced a lower TNF-α and IL-6 cytokine production than P2C but much higher than P3C and Lyso (Fig. 2E to G).

### Bacterial lipopeptides induced an elevated fermentation.

**FIG 3:**
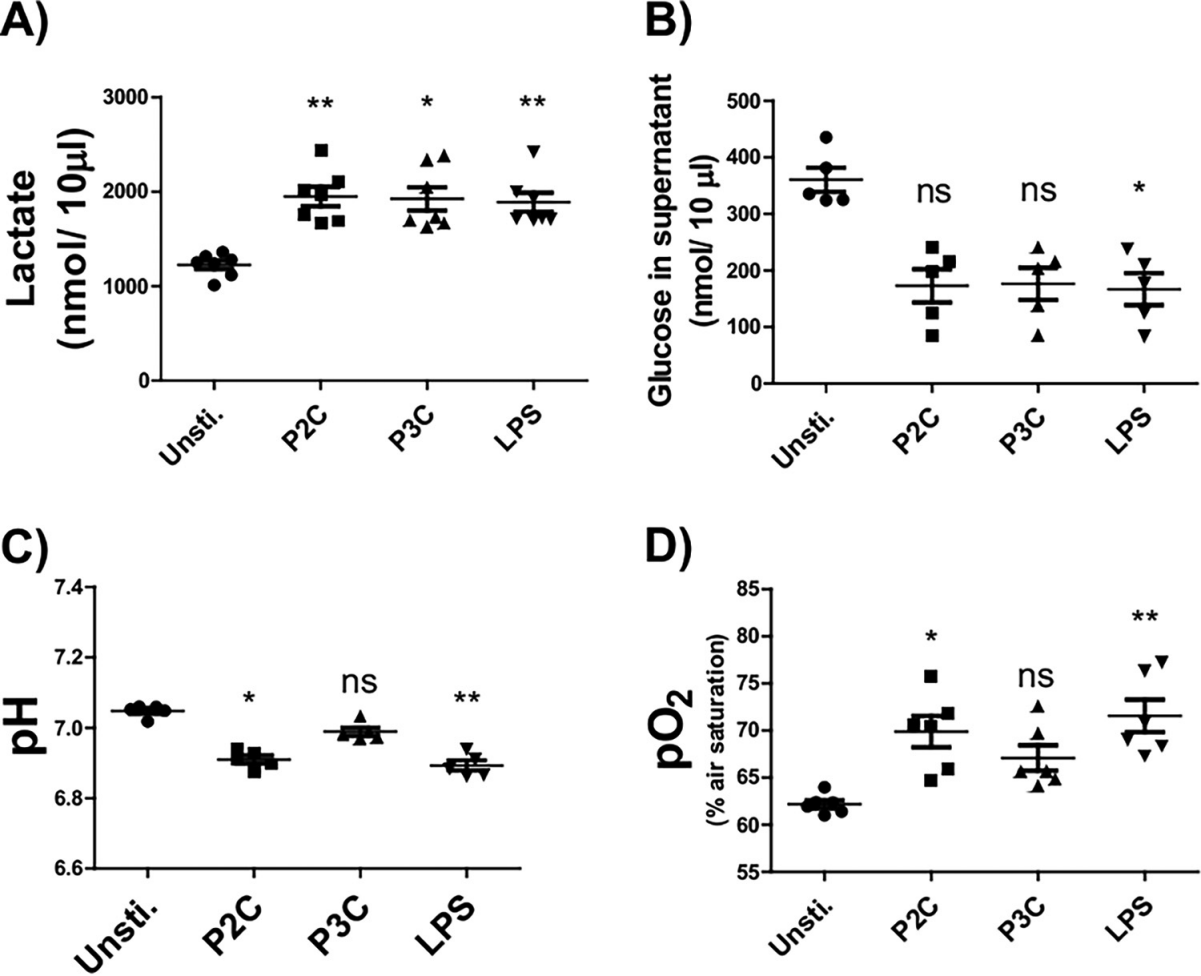
LPP analogues P2C and P3C confer significant effects on lactate, glucose consumption, pH, and oxygen consumption in BMDM. Experimental conditions were as described in Materials and Methods, and the various assessments such as Lactate (A), Glucose (B), pH (C) and pO_2_ (D) were performed in the supernatants of BMDM cells stimulated with 100 ng/mL of P2C, P3C, Lyso, or LPS, respectively, for 24 h in M199 medium. Control samples (unsti.) were used without adding any stimulators. The data were obtained from cells isolated from 5 to 6 mice. Error bars indicate mean ± SEM. Statistical significances were calculated between the treated cells and control (unsti.) by one-way ANOVA using Kruskal-Wallis comparison test: *, *P ≤ *0.05; **, *P < *0.01; ***, *P < *0.001.

In order to determine a putative effect of Lpp analogue treatment of BMDM and MM6 cells on the eukaryotic cell metabolism, we first determined the lactate concentration in the medium of BMDM after 24 h stimulation with P2C, P3C, and LPS. Supernatant lactate levels were found to be significantly elevated upon BMDM treatment with all three Lpp analogues compared to untreated cells (Fig. 3A). Moreover, a strong reduction of the glucose concentration in the supernatant was observed in all treated samples, yet this difference was only significant in the LPS-treated sample (Fig. 3B). Finally, a significant decrease of the pH, as well as a reduced consumption of oxygen, albeit only in P2C- and LPS-treated cells, was observed (Fig. 3C and D). Corresponding experiments were also carried out in MM6 cells; the results are presented in Fig. S2 and support our assumption, namely, that a significant metabolic shift toward fermentation also occurs in the P2C- and LPS-treated MM6 cells (Fig. S2A to F). The real-time oxygen concentration values are presented in Fig. S3.

**FIG 4:**
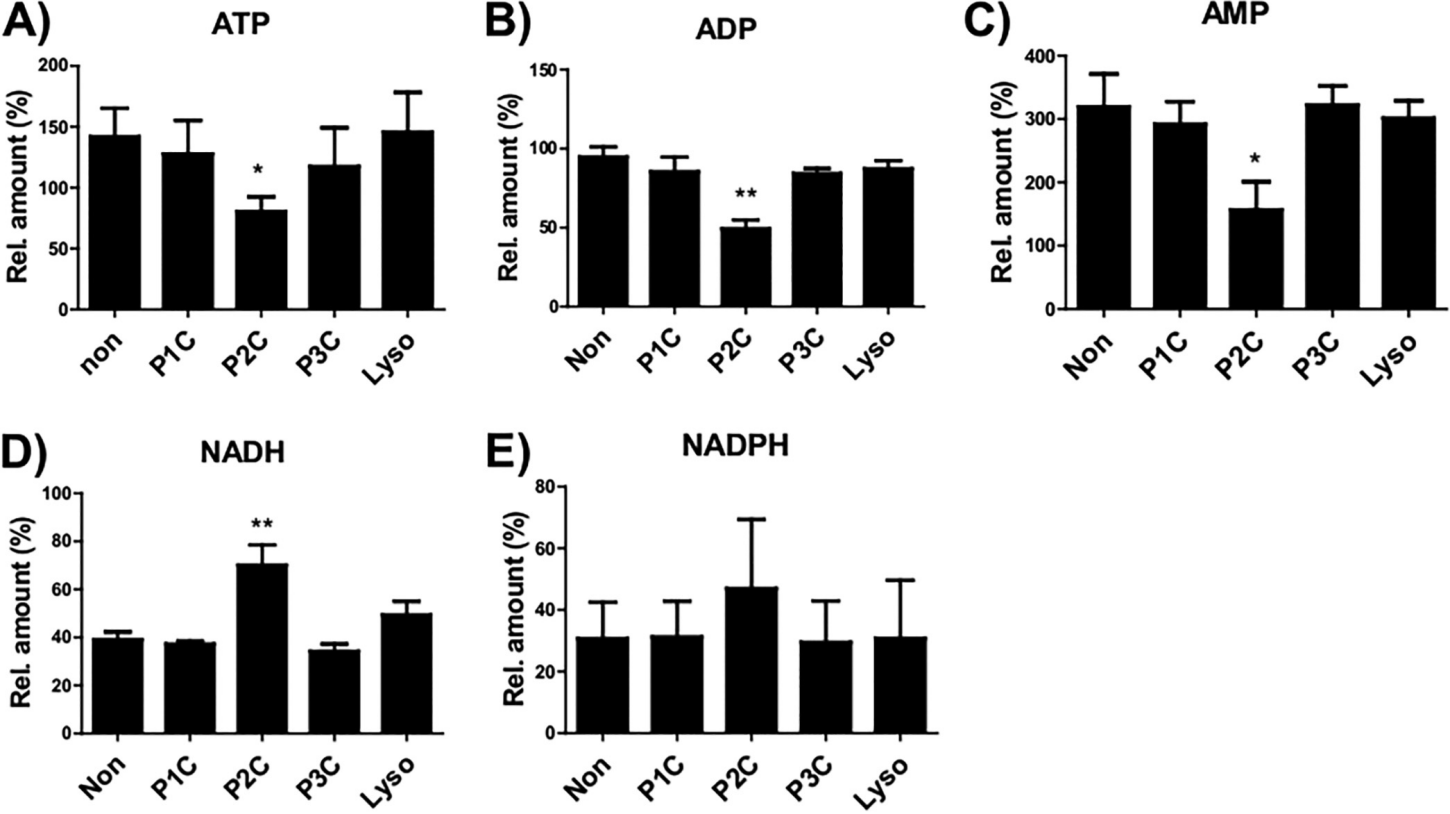
Metabolite analysis of MM6 treated with lipopeptides via capillary IC-MS. 5 × 10^6^ MM6 cells with indicated LPP analogues for 24 h. Control samples (non) were used without adding any stimulators. Metabolites including ATP (A), ADP (B), AMP (C), NADH (D) and NADPH (E) were determined by accurate mass and retention time by comparing them to authentic standards. The relative numbers of metabolites were calculated by comparing them to the internal standard ^13^C_2_-labeled succinic acid (as 100%). The data present the average values from 3 independent experiments. Error bars indicate SEM. Statistical significances were calculated between the treated cells compared to control (non) by using the analysis of 1-tailed Student’s *t* test: *, *P ≤ *0.05; **, *P < *0.01.

Next, we used capillary ion chromatography-mass spectrometry (IC-MS) employing a protocol optimized for MM6 cell determination of the number of cellular energy metabolites. Again, measurements were performed upon stimulation with P1C, P2C, P3C, and Lyso for 24 h. The mass spectrometric data for the extracts indicated that ATP, ADP, and AMP levels were significantly reduced in the cells treated with P2C compared to untreated cells (Fig. 4A to C). Moreover, the data show that the exposure to P1C, P3C, and Lyso did not induce similar effects. Along with these results, elevated NADH and NADPH levels were observed in P2C-treated cells compared to untreated cells (Fig. 4D and E).

These data suggested that a high level of TLR2 activation induced by an elevated cytokine production might cause major metabolism rewiring. Hence, to evaluate the impact of released cytokines on metabolic shift, the released cytokines were inactivated by using mouse neutralizing antibodies against TNF-α, IL-6, and IL-10. As a result, Lpl1(+sp) and P2C still caused lactate accumulation, glucose consumption, and pH and oxygen consumption in BMDMs regardless of whether or not in the presence of anti-mouse TNF-α, IL-6, and IL-10 antibodies (Fig. S4). In MM6 cells, the same observation was obtained when the released cytokines were inactivated by using anti-human neutralizing antibodies against TNF-α, IL-6, and IL-10 (Fig. S5). Taken together, these data demonstrate that other TLR2-activated components, but not these checked cytokines, have an impact on metabolic shift in monocytes/macrophages caused by Lpl1(+sp) or P2C.

### P2C, but not P3C, caused bone erosion in the mouse model.

**FIG 5:**
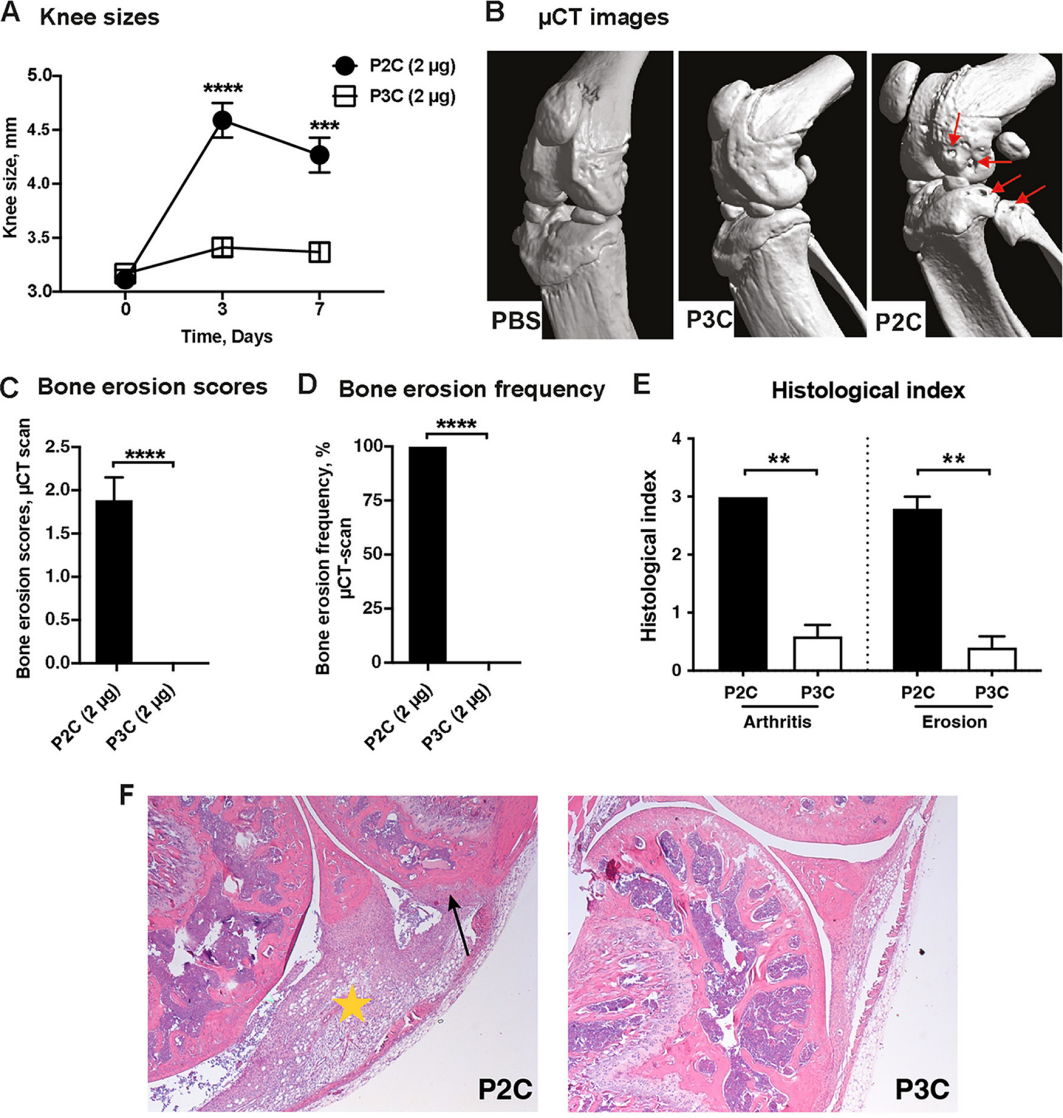
P2C induces bone erosion. (A) Measurement of knee swelling (mm) of NMRI mice (*n* = 9/group) up to 7 days after i.a. injection with 20 μL (2 μg) of P2C or P3C. (B) Representative microcomputed tomography (μCT) images of mice knee joints i.a. injected with phosphate-buffered saline (PBS) as a healthy control (left), P3C (middle), or P2C (right) on day 7 postinjection (arrows indicate bone erosion in the knee joint). (C and D) Bone erosion scores (C) and bone erosion frequency (D) of mice knee joints procured through scanning of joints in a μCT scanner 7 days after i.a. injection with P2C or P3C. Data were pooled from two independent experiments. (E) Histological evaluation of the knee joints from NMRI mice i.a. injected with 20 μL (2 μg) of P2C or P3C on day 7 postinjection. (F) Representative photomicrographs of knee joints of NMRI mice receiving 20 μL (2 μg) of P2C or P3C on day 7 postinjection, stained with hematoxylin and eosin. Arrow indicates bone erosion, and star indicates inflamed synovium. Statistical evaluations were performed using the Mann-Whitney *U* test, with data expressed as the mean with SEM (A, C, and E) or Fisher’s exact test (D); **, *P* < 0.01; ***, *P* < 0.001; ****, *P* < 0.0001.

**FIG 6:**
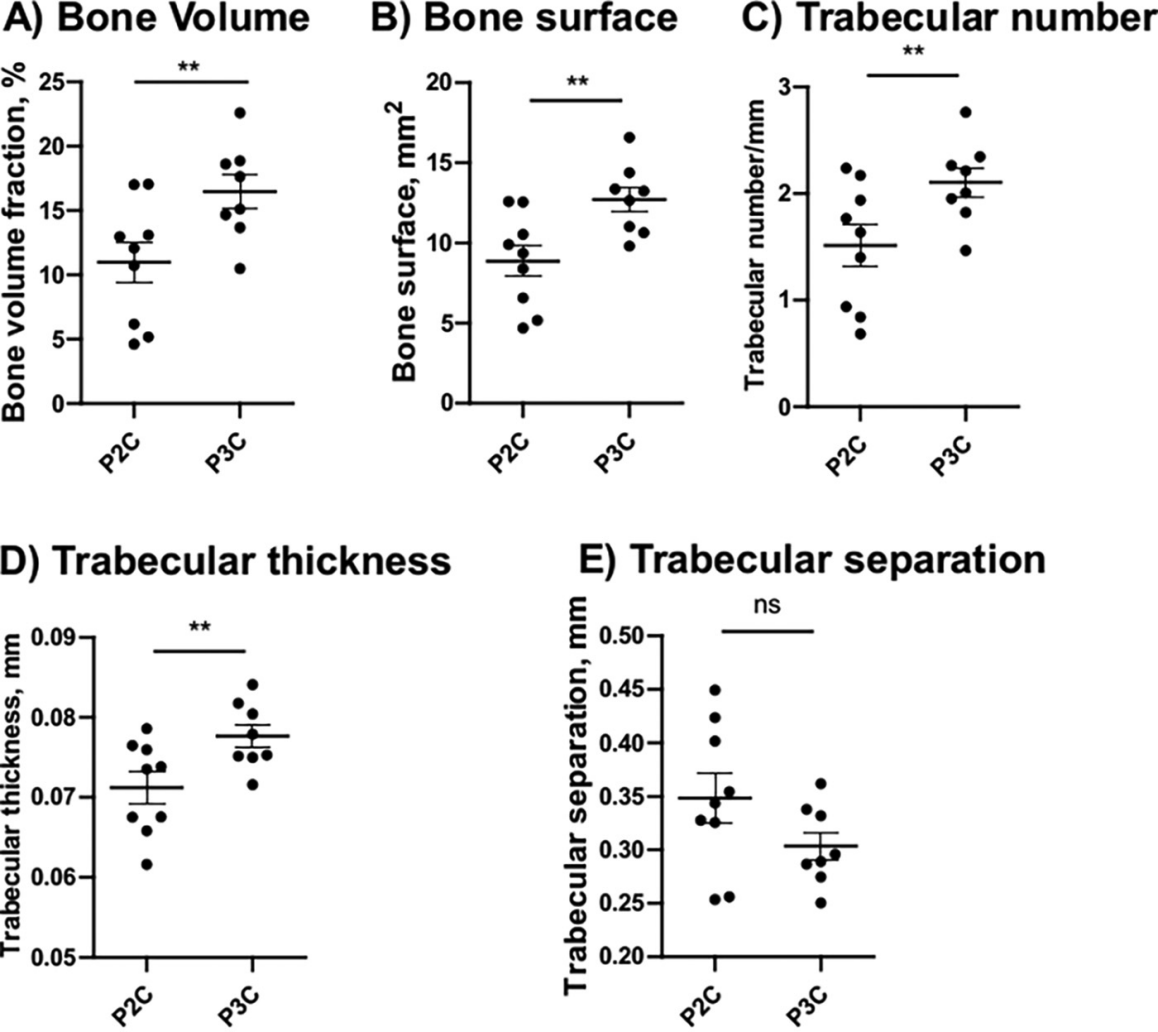
P2C is more potent in inducing bone resorption than P3C. (A to E) The trabecular bone volume fraction (A), bone surface (B), trabecular number per millimeter (C), trabecular thickness (D), and trabecular separation (E) were measured in NMRI mice (*n* = 9/group) up to 7 days after i.a. injection with 20 μL (2 μg) of P2C or P3C. (B) Representative microcomputed tomography (μCT) images of mice knee joints i.a. injected with P2C or P3C on day 7 postinjection. Data were pooled from two independent experiments. Statistical evaluations were performed using the Mann-Whitney U test. The data are presented as a scatterplot with data expressed as the mean ± SEM (*, *P* ≤ 0.05; **, *P* < 0.01; ns, not significant).

Mice knee joints were injected intra-articularly (i.a.) with 2 μg of either P2C or P3C. Intriguingly, significantly more knee swellings were found in joints injected with P2C than P3C on day 3 post i.a. injection, with substantial differences lasting until day 7 (Fig. 5A). Similarly, bone erosion scores and frequency values acquired through a microcomputed tomography (μCT) scan, as well as the histological analyses (arthritis scores and bone erosion scores) after hematoxylin and eosin (H&E) staining on day 7 postinjection, were observed to be significantly higher in joints injected with P2C than with P3C (Fig. 5C to E). Our data demonstrate that P2C was more potent in inducing bone resorption than P3C (Fig. 6). Figure 5B shows representative images of radiological signs of bone destruction by μCT, and Fig. 5F shows representative images of histological signs of joint inflammation and bone destruction in mouse joints i.a. injected with P2C and P3C on day 7 after injection.

### The lipopeptide-induced lactate and malate accumulation in knee joints requires the presence of macrophages.

**FIG 7:**
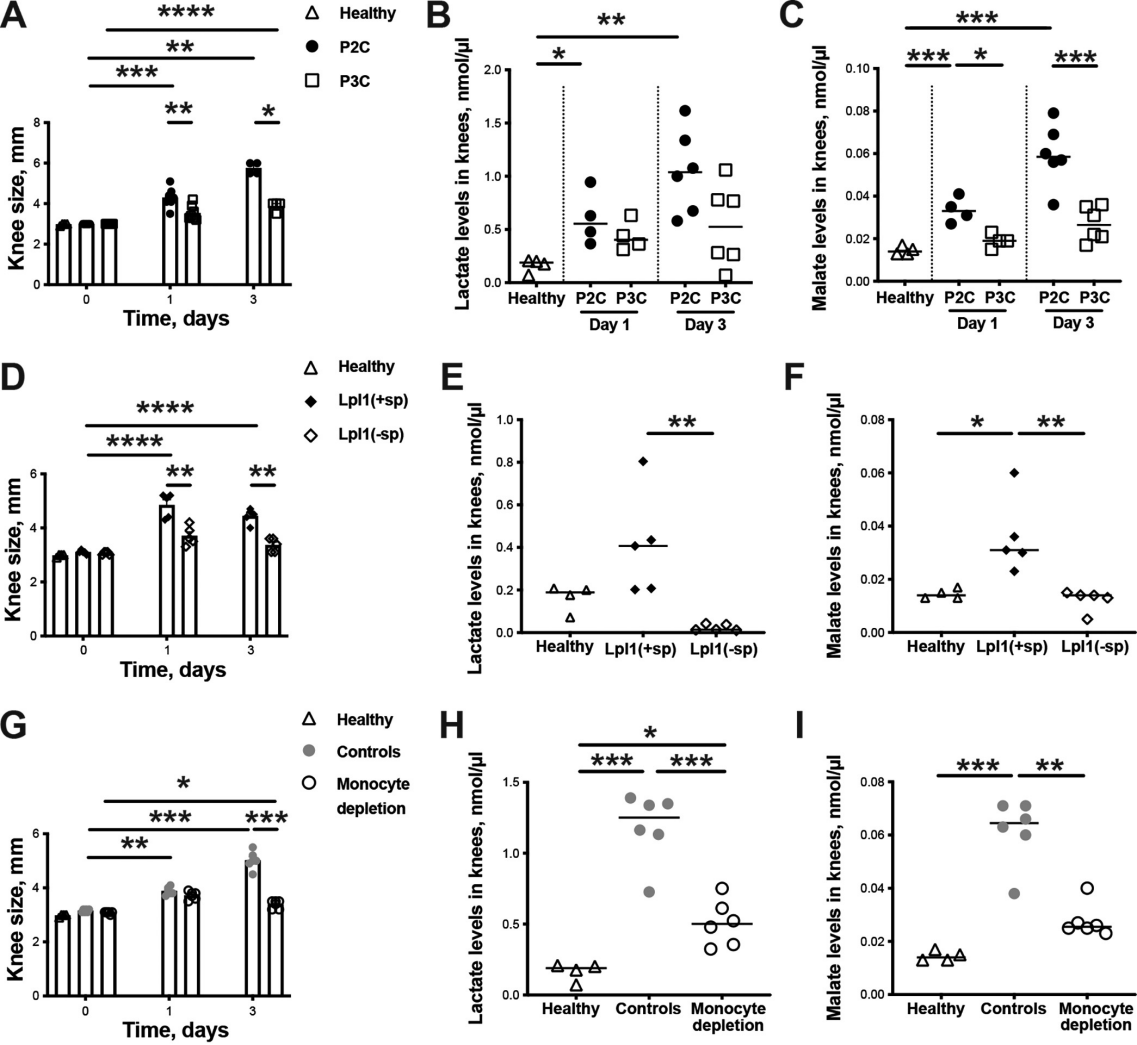
P2C upregulates lactate expression in local knee joints of mice through monocytes/macrophages. (A) Measurement of knee swelling (mm) of NMRI mice up to 3 days after i.a. injection with 20 μL of phosphate-buffered saline (PBS) (healthy; *n* = 4) or the synthetic lipopeptides, P2C or P3C (4 μg/knee; *n* = 4 to 6/group). (B and C) Levels of lactate and malate, respectively, in the supernatant from knee homogenates of NMRI mice on day 1 and on day 3 after i.a. injection with 20 μL of PBS (healthy; *n* = 4) or the synthetic lipopeptides, P2C or P3C (4 μg/knee; *n* = 4 to 6/group). (D) Measurement of knee swelling (mm) of NMRI mice up to 3 days after i.a. injection with 20 μL of phosphate-buffered saline (PBS) (healthy; *n* = 4), Lpl1(+sp), or Lpl1(−sp) (4 μg/knee; *n* = 5/group). (E and F) Levels of lactate (E) and malate (F) in the supernatant from knee homogenates of NMRI mice on day 3 after i.a. injection with 20 μL of PBS (healthy; *n* = 4), or Lpl1(+sp) or Lpl1(−sp) (4 μg/knee; *n* = 5/group). (G) Measurement of knee swelling (mm) of NMRI mice depleted of monocytes/macrophages using clodronate liposomes up to 3 days after i.a. injection with 20 μL of P2C (4 μg/knee; *n* = 6/group). (H and I) Levels of lactate (H) and malate (I) in the supernatant from knee homogenates of NMRI mice depleted of monocytes/macrophages using clodronate liposomes on day 3 after i.a. injection with 20 μL of P2C (4 μg/knee; *n* = 6/group). Data were pooled from two independent experiments. Statistical evaluations were performed using one-way analysis of variance (ANOVA) with Tukey's test or Kruskal-Wallis test with Dunn’s multiple-comparison test, with data expressed as the mean with SEM (A, D, and G) or the median (B, C, E, F, H, and I); *, *P ≤ *0.05; **, *P < *0.01; ***, *P* < 0.001; ****, *P* < 0.0001.

To investigate whether P2C or P3C induce local lactate expression, 4 μg of either P2C or P3C were i.a. injected into local knee joints of mice. On day 1 and on day 3 postinjection, the knee joints of healthy mice and P2C- or P3C-injected mice were collected and assessed. As expected, more pronounced knee swellings were found in joints injected with P2C than in P3C-injected joints or, to healthy controls, both on day 1 and day 3 after injection (Fig. 7A). Interestingly, the lactate levels were upregulated in the P2C-injected knee joints compared to healthy controls at both time points, whereas P3C-injected joints did not display significant higher lactate levels than healthy controls (Fig. 7B), suggesting that the di-acylated LPP analogue structure is more potent than the tri-acylated LPP structure in causing the lactate accumulation. Also, P2C induced significantly higher malate levels in local joints than P3C on both day 1 and day 3, although the malate levels were relatively low compared to lactate in all samples (Fig. 7C).

To further understand whether staphylococcal lipoproteins can induce the metabolites in joints, we injected Lpl1(+sp) and Lpl1(−sp) into mouse knees. Lpl1(+sp), but not Lpl1(−sp), caused knee swelling (Fig. 7D). Importantly, Lpl1(+sp), but not Lpl1(−sp), induced higher levels of lactate and malate (Fig. 7E and F), suggesting that S. aureus lipoproteins can induce these metabolites in inflamed joints.

To evaluate the role of macrophages in lactate accumulation in mice injected with P2C, we used the monocyte/macrophage-depleted mouse model for P2C i.a. knee joint injection with regard to the arthritogenic properties and lactate expression. Indeed, depletion of infiltrating monocytes and synovial macrophages by clodronate liposomes significantly reduced the macroscopic joint inflammation, as well as the lactate and malate levels, on day 3 after injection (Fig. 7G to I). Of note, fumarate levels were also investigated, but the value was under the detectable limit in the knee homogenates.

## DISCUSSION

The mechanism of how a bacterial membrane constituent nontoxic to mammalian cells, i.e., the purified S. aureus lipoprotein Lpl1(+sp), confers gross destruction of bone in a mouse arthritis model remained a conundrum after completion of our previous study (6). Involvement of the native immune system, however, was suggested in this work upon the observation that the observed bone destruction was dependent on the presence of macrophages in the model. With ample evidence, in the here-presented study, these open questions could now be addressed by the demonstration of TLR2 activation by P2C, resulting in a profound shift of metabolism in bone marrow-derived macrophages as well as in the well-established MM6 line.

Different lipopeptides (P2C, P3C, and Lyso), which mimic Lpps, could trigger the production of cytokines, albeit to a different extent. The di-acylated lipopeptides (P2C) that were shown to stir a more pronounced cytokine response both in BMDMs and MM6 than in the other Lpp analogues and control caused a profound switch of the preferred metabolic pathway, as evidenced by analysis of glycolytic/respiration activity readouts. In contrast, the other lipopeptides (P3C and Lyso), inducing lower levels of cytokines, conferred a significantly lower impact on metabolic alteration.

The impact of Lpp structures on host cytokine response and inflammation has already been demonstrated by others both *in vitro* and *in vivo*, revealing that P2C triggers a starker cytokine response than P3C and Lyso (4, 5, 26–28). While we could confirm these results when employing BMDMs, here, we also show that this effect is not paralleled in other types of immune cells, for example, bone marrow monocytes (Fig. 2C and D) and human PBMCs and monocytes (29). Therefore, TLR2-conferred immune cell activation is more complicated than had been previously considered.

The number of previous studies analyzing the impact of bacterial Lpps and lipopeptides on host cellular metabolism is limited. In a study by Lachmandas et al., the authors demonstrated that there is no observation of the shift from oxidative phosphorylation (OXPHOS) to glycolysis in monocytes stimulated with P3C (30). Stimulation of rheumatoid arthritis synovial fibroblast cells (RASFCs) with high concentrations of P3C (1 μg/mL) caused induction of PKM2 nuclear translocation, inhibition of ATP synthesis, and enhanced glycolysis (31). These data suggested that a high level of TLR2 activation induced by an elevated cytokine production caused major metabolism rewiring. However, our data suggest that not the major cytokines we checked, yet other hitherto unidentified factors in the TLR2-dependent cell activation pathway appear to be responsible for triggering the metabolic reprogramming. Recently, Balic and colleagues reported that STAT3 phosphorylation is the crucial signaling intermediary for TLR4-induced glycolysis (32). It has been shown in previous studies that TNF and IL-6 were responsible for the formation of osteoclast-like cells, which mediated bone loss (33–36). However, our *in vitro* data indicate that these cytokines are likely not responsible for the metabolic shift of macrophages triggered by bacterial lipoproteins. In this study, we present compelling evidence that establishes a subtle connection between bone erosion and the increased metabolites resulting from the fermentative metabolism of macrophages in inflamed joints, while it remains possible that both inflammatory cytokines and metabolic shifts contribute to bone destruction in septic arthritis through distinct pathways or mechanisms.

It has been demonstrated that the activation of macrophages and dendritic cells by proinflammatory stimuli leads to a switch from OXPHOS to the glycolysis pathway even under aerobic conditions, similar to the Warburg effect ascribed to the altered metabolism observed in cancer cells (37). Similar to these observations, P2C-stimulated BMDMs conferred a switch of metabolism toward a fermentative pathway with a lower oxygen consumption and a larger amount of accumulated lactate, resulting in reduced pH levels. Indeed, higher levels of lactate (38) and lower levels of glucose (39) have been described in synovial fluids from septic arthritis versus arthritis caused by other inflammatory mechanisms or by degenerative joint disease. Our *in vitro* findings closely relate to the observation of *in vivo* experiments, as, conceivably, the lower pH, decreased by lactate accumulation induced by Lpl1(+sp)-P2C-stimulated monocyte/macrophage cells *in vivo*, leads to the bone erosions in mouse joints (Fig. 5 and 6). It has been demonstrated that extracellular pH directly regulates bone cell response, and indeed, a low pH, around 6.9, is required for osteoclasts to resorb the bone matrix, whereas resorption is drastically reduced at pH above 7.4 (40, 41). In addition, secretion of acids by osteoclasts is known to play a central role in both systemic and focal bone resorption (42). Our experiment with monocyte/macrophage-depleted mice has demonstrated that macrophages were the main responsible factors for inflammation and lactate accumulation in mouse joints infected with P2C and subsequently resulted in bone erosion.

In dendritic cells, macrophages, and monocytes, the engagement of LPS with TLR4 and the ensuing receptor stimulation results in higher glucose consumption and leads to the accumulation of tricarboxylic acid (TCA) intermediates such as citrate, succinate, fumarate, and malate (30, 43, 44). Besides lactate, we also observed a higher level of malate in murine joint injected with Lpl1(+sp) and P2C, but not in monocyte/macrophage-depleted cells. Both TLR2 and TLR4 activation were shown to result in elevated NADH, an important cofactor for diverse biosynthesis intermediates supporting anabolic growth (9).

Previous studies have shown that LPP-activated TLR2 induced activation and apoptosis in THP1- and TLR2-transfected HEK293 cells (45). However, in our study, LPP analogue-activated TLR2 did not trigger apoptosis or necrosis in MM6 cells (see Fig. S6 in the supplemental material). This result strongly suggests that the altered ATP, ADP, AMP, and TCA intermediate levels in TLR2-activated MM6 cells were caused by the metabolic shift, yet not by apoptosis or necrosis.

In addition, S. aureus is known to release protein A (SpA), which, bound to osteoblasts, enhances the secretion of soluble RANKL, resulting in activation of bone-resorbing osteoclasts (46). RANKL is a 317-amino-acid polypeptide belonging to the tumor necrosis factor (TNF) family and binding to the RANK receptor (46). Besides mediating osteoclastogenesis and bone resorption, RANKL/RANK signaling was shown to be involved in dendritic cell survival and function, macrophage activation, and T-cell differentiation and activation (47, 48). Whether or not the RANK/RANKL axis may be involved in the observed effects on LPP-dependent bone resorption is an interesting aspect for further studies.

### Conclusion.

The impact of metabolic reprogramming on cellular physiology and immune activation has recently stirred intense interest. Previous studies have shown that the immunometabolic response is specific for cell types (monocytes or macrophages), host species (murine or human), as well as bacterial stimuli (LPS as TLR4 ligands or LPPs as TLR2 ligands). It is therefore intriguing to speculate that a differential and fine-tuned cellular response to the various species of LPP underlies a programmed, evolutionary adaptation of the host to the challenge of a broad spectrum of immune modulators produced by microorganisms, from bacteria up to parasites.

Our data demonstrate that metabolic shift in macrophages mediates bone erosion caused by bacterial LPPs. The shift toward fermentation was indicated by decreased oxygen consumption, accumulated lactate, and a decreased pH, with potentially potent local consequences on bone and cartilage tissue but also on the cellular homeostasis at other infected body sites. Both the proinflammatory, as well as the metabolic, effects of LPPs on important members of the cellular immune system suggest a potential clinical use of our findings, e.g., sophisticated diagnostics through LPP analyses of the invading pathogen, anti-LPP, or LPP-analogue intervention strategies to modulate immune and tissue responses in clinical disease or targeting macrophage metabolic shift to prevent joint damage in septic arthritis.

## MATERIALS AND METHODS

### Ethics statement.

The Ethics Committee of Animal Research of Gothenburg approved all experiments conducted on mice. The mouse experiments were performed in accordance with the Swedish Board of Agriculture's regulations and recommendations on animal experiments. The allowance to kill the mice to obtain the mouse bone marrow cells was approved by State Office for Nature, Environment and Consumer Protection (LANUV), North Rhine-Westphalia (approval number 81-02.05.50.22.004). The experiments were performed following the §4(3) TierSchG regulation.

### Mice.

Eight- to 12-week-old female healthy NMRI mice were purchased from Envigo (Venray, Netherlands) and stored under standard temperature, light, and nutrition conditions (10 mice/cage) in the animal facility of the University of Gothenburg. For obtaining the mouse bone marrow cells, 8- to 10-week-old male and female C57BL/6 mice were purchased from the Central Animal Experimental Facility (ZTE), University of Münster, and kept in the animal facility of the Institute of Medical Microbiology, University Hospital of Münster.

### Chemicals and reagents.

P1C (Pam-DHC-CSK4) is a synthetic monopalmitoylated lipopeptide. P2C (Pam_2_CSK_4_) is a synthetic dipalmitoylated lipopeptide that mimics the di-acylated amino terminus of LPP. P3C (Pam_3_CSK_4_) is a synthetic dipalmitoylated *N*-palmitoylated lipopeptide that mimics the tri-acylated amino terminus of LPP, and Lyso (PamCysPamCSK_4_), a synthetic monopalmitoylated *N*-palmitoylated lipopeptide, mimics the Lyso-acylated amino terminus of LPP. All these peptides were chemically synthesized by EMC (Tübingen, Germany). The chemical structures of these peptides are shown in Fig. S1 in the supplemental material.

Lipopolysaccharide (LPS-B5 Ultrapure) was purchased from InvivoGen (catalog no. tlrl-pb5lps; InvivoGen, Germany). Human cytokines TNF-α (catalog no. T6674), IL-6 (catalog no. SCU0001), IL-10 (catalog no. H7541), and succinic acid-2,3-^13^C_2_ (catalog no. 488364) were purchased from Sigma (Germany).

### Expression and Purification of Lpl1(+sp) and Lpl1 (−sp).

S. aureus lipoproteins Lpl1(+sp) (plus signal peptide) and Lpl1(−sp) (minus signal peptide) were prepared and purified as previously described (28). Lpl1(+sp) expressed with lipid moiety was extracted from the membrane fraction of SA113 [pTX30::lpl1(+sp)-his], while the truncated Lpl1(−sp) without lipid moiety was extracted from the cytoplasmic fraction of SA113 Δ*lgt* [pTX30::lpl1(−sp)-his]. The Lpl1s were concentrated via a centrifugal ultrafilter unit with a molecular mass cutoff of 10 kDa (Sartorius AG, Göttingen, Germany). The purification and the concentration of Lpl1(+sp) and Lpl1(−sp) were confirmed by SDS-PAGE. One milligram per milliliter of purified compounds of Lpl1 was aliquoted and stored at −70°C until use and was adjusted with phosphate-buffered saline (PBS) buffer to obtain the required concentration before the experiment.

### Mouse bone marrow monocyte cell and bone marrow-derived macrophage isolation.

Bone marrow cells were isolated from murine femur and tibiae. Erythrocytes were depleted by osmotic shock, and cells were washed and collected by centrifugation. Mouse bone marrow monocytes were isolated from bone marrow cells by using monocyte isolation kit (BM) (catalog no. 130-100-629; Miltenyi Biotec, Bergisch-Gladbach, Germany). The mouse bone marrow monocytes were resuspended with RPMI medium supplemented with 10% fetal calf serum (FCS) and 1% penicillin-streptomycin for the stimulation test. To generate the BMDMs, bone marrow cells were cultured in a 6-well Cellstar cell culture plate (catalog no. 657970; Greiner Bio-One, Frickenhausen, Germany) with a concentration of 10^6^ cells/mL of M199 medium supplemented with 10% FCS and 1% penicillin-streptomycin. We added 100 ng/mL macrophage colony-stimulating factor (M-CSF) (catalog no. 130-101-700; Miltenyi Biotec, Bergisch-Gladbach, Germany) on day 0 and day 3. The BMDMs were harvested after 7 days and resuspended in the fresh M199 medium supplemented with 10% FCS and 1% penicillin-streptomycin for the stimulation test.

### Stimulation assay.

Mouse bone marrow monocytes and BMDMs were seeded in the RPMI medium and M199 medium, respectively, with a concentration of 10^6^ cells/mL for the stimulation. We stimulated 1 × 10^5^ cells/100 μL/well in 96-well cell culture plates with 300 ng/mL of Lpl1(+sp) and Lpl1(−sp) or 100 ng/mL of P2C, P3C, or LPS. The cell cultures were incubated for 20 h to measure mTNF-α and mIL-6. Supernatants were collected and stored at −20°C until they were used for enzyme-linked immunosorbent assay (ELISA). Lactate, glucose, oxygen, and pH measurements were performed after 24 h of incubation.

MonoMac 6 (MM6), a human monocyte-macrophage cell lineage, was obtained from DSMZ (AAC 124, Braunschweig, Germany) and cultured as previously described with medium (RPMI, 10% FCS, 1% OPI in nonessential amino acids (NEA), 1% penicillin-streptomycin, and 1% l-glutamine) (28). For the stimulation assay, MM6 cells were seeded 5 × 10^4^ cells/100 μL/well into 96-well cell culture plates and incubated for 1 h at 37°C with 5% CO_2_. For stimulation, 100 μL of medium containing P1C, P2C, P3C, Lyso, and LPS was added to the cells to obtain 200 μL volume with the final concentrations of 50 ng, 100 ng, 250 ng, and 500 ng/mL, respectively. The cell cultures were incubated for 5 h to measure hTNF-α and for 24 h to measure hIL-6 and hIL-10. Supernatants were collected and stored at −20°C until they were used for ELISA. To examine the Warburg effect, ATP levels were measured after 72 h of incubation, while lactate, glucose, oxygen, and pH measurements were performed after 24 h of incubation.

### Antibody-dependent neutralization of TNF-α, IL-6, and IL-10 cytokines.

For blocking assay in BMDM cells, 5 × 10^5^ cells were incubated for 1 h with either 1 μg/mL of mouse TNF-α neutralizing rabbit antibodies (catalog no. D2H4; Cell Signaling, Leiden, Netherlands), 1 μg/mL of mouse IL-6 neutralizing mouse antibodies (catalog no. mil6-mab15-02; InvivoGen, Toulouse, France),1 μg/mL of mouse IL-10 neutralizing rat antibodies (catalog no. 16-7102-81; Thermo Fisher, Schwerte, Germany), or 1 μg/mL mouse anti-mouse monoclonal antibody IgG1 (catalog no. mabg1-ctrlm; InvivoGen, Toulouse, France) as isotype control. After the blocking process, the cells were incubated with 300 ng/mL of Lpl1(+sp) or 100 ng/mL P2C for 24 h for further analysis.

For the blocking assay in MM6 cells, 5 × 10^5^ cells were incubated for 1 h with either 1 μg/mL of rabbit anti-human antibodies TNF (catalog no. D1B4; Cell Signaling, Leiden, Netherlands), 1 μg/mL of mouse anti-human antibody IL-6 (catalog no. mabg-hil6-3; InvivoGen, Toulouse, France), 1 μg/mL of rat antihuman antibody IL-10 (catalog no. AHC0103; Thermo Fisher, Schwerte, Germany), or 1 μg/mL mouse anti-human monoclonal antibody IgG1 (catalog no. bgal-mab1-ctrlm; InvivoGen, Toulouse, France) as isotype control. After the blocking process, the cells were incubated with 100 ng/mL P2C for 24 h for further analysis.

### ELISA.

Mouse and human cytokine secretion was measured in cellular supernatants using the Invitrogen eBioscience ELISA Set Go kits (Fisher Scientific, Schwerte, Germany) for mouse cytokines (TNF-α and IL-6) and human cytokines (TNF-α, IL-6, and IL-10) according to the manufacturer’s instructions.

### The Warburg effect.

After 72 h, stimulated MM6 cultures were measured by qualifying the optical density (*A*_570_) and calculating the Warburg effect as 1/*A*_570_ (49, 50). In detail, the Warburg effect was determined based on the change in the color of organic pH indicators in the medium (51). The optical density values were measured by the Synergy HTX multimode reader (BioTek, Bad Friedrichshall, Germany).

### ATP assay.

The 72-h-stimulated MM6 cells collected from 4 wells (around 2 × 10^5^ cells) were harvested by centrifugation (2,000 rpm, 1 min, room temperature [RT]) and suspended in 200 μL sterile Milli-Q water. The cell suspensions were vortexed shortly and then boiled at 99°C for 6 min and vortexed a second time for 10 s. The lysate suspensions were centrifuged for 1 min at 14,000 rpm and RT. Subsequently, 20-μL lysate supernatants were diluted with 30 μL ATP assay buffer to obtain 50 μL volume in a black 96-well plate for ATP fluorometric assay kit (catalog no. MAK190; Sigma, Germany). The fluorescence was measured using the Synergy HTX multimode reader with an excitation filter of 530/25 nm and an emission filter of 620/40 nm.

### Lactate assays.

The 24-h-stimulated cell supernatants were collected by centrifugation (2,000 rpm, 1 min, RT) and diluted 20 times with a lactate assay buffer (catalog no. MAK064; Sigma, Germany). Fifty microliters of the diluted supernatant was added into a transparent 96-well plate prepared for determination using the lactate colorimetric assay kit (catalog no. MAK064; Sigma, Germany). Absorbance was measured at 570 nm with a Synergy HTX multimode reader.

### Glucose assay.

The 24-h-simulated cell supernatants were collected by centrifugation (2,000 rpm, 1 min, RT) and then diluted 10 times with glucose assay buffer (catalog no. MAK263; Sigma, Germany). Ten microliters of the diluted supernatant was added to 40 μL glucose assay buffer to obtain 50 μL volume in a transparent 96-well plate for the glucose colorimetric assay kit (catalog no. MAK263; Sigma, Germany). The absorbance was measured at 570 nm with Synergy HTX multimode reader.

### Oxygen and pH measurement.

The real-time oxygen concentration and the pH in the medium were monitored by SensorDish reader (Presens, Germany). In detail, 1 mL containing 5 × 10^5^ cells was stimulated with 100 ng/mL of P1C, P2C, P3C, Lyso, or LPS in a 24-well OxoDish plate to monitor oxygen concentration or in a 24-well HydroDish plate to monitor pH. The sensor contains an oxygen- or pH-sensitive indicator dye immobilized in a thin polymer film. The oxygen and pH values were recorded every hour for 24 h.

### Apoptosis necrosis assay.

**(i) FITC annexin V apoptosis detection.** We incubated 5 × 10^4^ MM6 cells with 100 ng of P1C, P2C, P3C, or Lyso for 24 h, and they were washed twice with cold phosphate-buffered saline (PBS) and resuspended in 100 μL of binding buffer. Subsequently, the cells were stained with 2 μL of fluorescein isothiocyanate (FITC)-annexin V or 4 μL propidium iodide (PI) for 15 min at RT in the dark according to the annexin V apoptosis detection kit (BD Pharmigen, Heidelberg, Germany). Then, 400 μL of binding buffer was added into each tube, and cells were analyzed by flow cytometry (BD Accuri C6). The cells incubated with medium containing 10% dimethyl sulfoxide (DMSO) were used as positive apoptosis control. Data were analyzed with BD Accuri C6 software.

**(ii) RealTime-Glo annexin V apoptosis and necrosis assay.** Apoptosis and necrosis assay were further detected as a function of incubation time by using the RealTime-Glo annexin V kit (Promega). Briefly, 5 × 10^4^ MM6 cells were incubated with 100 ng of P1C, P2C, P3C, or Lyso in a white 96-well plate in RPMI medium supplied with detection reagent (catalog no. JA1011; Promega, USA). The stimulated cells were incubated at 37°C and 5% CO_2_ and were measured after 3, 5, and 24 h of incubation. Apoptosis readings were taken as relative luminescence units (RLU), and necrosis values were determined as RLUs at a wavelength of 485 ± 20 nm excitation and 525 ± 30 nm emission.

### Metabolite extraction and sample preparation.

MM6 cells were stimulated with 100 ng of P1C, P2C, P3C, or Lyso for 24 h or a control sample without any stimulator. For metabolite extraction, 5 × 10^4^ MM6 cells of each sample was harvested and washed twice with ice-cold PBS buffer. Cell pellets were suspended in 0.2 mL of ice-cold methanol and 0.1 mL of ultrapure water containing 10 μmol/L succinic acid-2,3-^13^C_2_ as internal standard (IS) and 0.5 mL chloroform and vortexed for 20 s. The suspension was shaken for 45 min at 750 rpm at 6°C. Then, 0.5 mL methanol and 0.15 mL IS water were added to each sample and shaken for another 15 min. Subsequently, the samples were centrifuged at 4°C and 14,000 × *g* for 10 min for layer separation. The aqueous layer containing metabolites was transferred into a new tube and stored at −80°C until analysis.

### Capillary IC-MS analysis.

Capillary IC-MS analysis was performed using a Dionex ICS-4000 capillary high-performance ion chromatography (HPIC) system connected to a Q Exactive Plus (Thermo Fisher Scientific, Dreieich, Germany) utilizing a heated ESI II (HESI-II) electrospray ionization (ESI) source. Sample injections were performed by a Dionex AS-AP autosampler, and for delivery of a regeneration water flow and a makeup solution flow, the system was equipped with two external AXP auxiliary pumps from Thermo Fisher Scientific (Dreieich, Germany).

Samples were separated on a Dionex IonPac AS11-HC-4 μm column (250 by 0.4 mm, 4 μm; Thermo Fisher Scientific) that was maintained at 35°C. The flow rate was set to 17 μL/min, and the injection volume was 0.4 μL. The KOH gradient program utilized for the separation was 1 mmol/L KOH held for 2 min, increased to 13 mmol/L at 8 min, 18 mmol/L at 12 min, 30 mmol/L at 24 min, and a final increase to 80 mmol/L at 37 min held for 6 min, followed by a 2-min decrease back to initial conditions, and then held for 5 min. The total analysis time was 50 min. For an improved ionization, an acetonitrile/water solution (1:1) containing 0.1% (vol/vol) ammonium hydroxide was delivered as makeup flow at a flow rate of 20 μL/min, combined with the eluent via a low dead volume mixing tee, and passed through a grounding union before entering the ESI source.

The Q Exactive mass spectrometer was operated in negative ionization mode, and the spray voltage was set to 2.8 kV. The capillary temperature was set to 250°C, the sheath gas flow rate was 20 (arbitrary units), the auxiliary gas flow rate was 10 (arbitrary units), the sweep gas flow rate was 0 (arbitrary units), and the S-lens level was set to 50. Full scan mode was utilized with the following parameters for the analysis of the cell extracts: resolution, 70,000 (full width at half-maximum, FWHM); auto gain control target, 5 × 10^5^; maximum ion injection time, 100 ms; and scan range in the mass range of *m/z* 70 to 750.

For IC-MS instrument control, Xcalibur 4.1 software and the SII plugin (Thermo Scientific) were utilized. Quantification of metabolites was performed on the basis of extracted ion chromatograms (relative mass deviation of ±10 ppm) of each compound for peak area determination by Xcalibur QualBrowser software. ^13^C-labeled succinic acid was used as internal standard. Metabolites were assigned by accurate mass (<5 ppm relative mass deviation) and retention times by comparison to authentic standards.

### Arthritis experiment *in vivo*.

To study the arthritogenic properties, as well as the lactate levels, in local knee joints of mice injected with the synthetic lipopeptides P2C and P3C, two sets of experiments were performed. One of the following materials was prepared in 20 μL of PBS and intra-articularly (i.a.) injected into the knee joints of NMRI mice: (i) 2 μg of P2C or P3C for assessment of the arthritogenic properties development of bone erosion, and (ii) 4 μg of P2C or P3C for assessment of the local expression of lactate levels. PBS served as control. The mice from the different treatment groups were mixed in the same cages to minimize potential confounders. The diameters of the knee joints of mice were measured by two observers (Z.H. and T.J.), blinded to the different treatment groups, with a caliper to determine the severity of the induced arthritis.

### *In vivo* cell depletion procedures.

In order to deplete both synovial residual macrophages as well as systemic monocytes, clodronate liposomes (Liposoma BV, Netherlands [52]) were utilized as the depletion procedure. NMRI mice received a volume of 20 μL i.a. into the knee joints and 200 μL intravenously (i.v.) for local and systemic depletion, respectively, 1 day before exposure to P2C. The i.v. treatment with 200 μL of clodronate liposomes continued 1 day after the P2C exposure. Similarly, PBS control liposomes (Liposoma BV, Netherlands) were injected into another set of mice in order to serve as controls, as previously described (6, 7).

### Homogenate preparation.

The knee joints, excluding the excessive surrounding tissues, were collected into individual Eppendorf tubes containing 1 mL of sterile PBS, followed by homogenization with TissueLyser II (Qiagen, Hilden, Germany). Afterward, the samples were centrifuged (11,000 rpm for 10 min at 4°C), and the supernatant was processed following metabolite extraction and sample preparation for assessment of metabolite expression. Lactate, fumarate, and malate were measured by using lactate assay kit (catalog no. MAK064; Sigma, Germany), fumarate assay kit (catalog no. MAK060, Sigma, Germany), and malate assay kit (catalog no. MAK067, Sigma, Germany) according to the manufacturer’s instructions.

### Microcomputed tomography.

Following the termination date of the experiments, the mouse knee joints were suspended in 4% formaldehyde for at least 7 days. To detect induced bone loss, mice knee joints were transferred to PBS and subsequently scanned *ex vivo* in the Skyscan 1176 μCT scanner (Bruker, Antwerp, Belgium). During scanning, the voltage and current were set to 55 kV and 455 μA, respectively, with a voxel size of 9 μm, an aluminum filter of 0.2 mm, and an exposure time of 755 ms. The scanning angular rotation was set to 180°, and the X-ray projections were retrieved at 0.42° intervals. To obtain three-dimensional reconstructions of P2C and P3C injected mice knee joints, the projection images were processed using NRecon software (version 1.6.9.8; Bruker) and analyzed with a CT analyzer (version 2.7.0; Bruker). Subsequently, the three-dimensionally reconstructed images were graded, on a grading scale from 0 to 3, by experienced observers (M.M. and T.J.) in a blinded manner for treatment groups on the extent of bone and cartilage destruction, as previously explained (53).

A morphometric analysis was performed on the distal femur bone. A 0.86-mm section of the distal femur was obtained from 100 micro-CT image slices, 50 slices above and below the reference point; the growth plate was selected to be the reference point. The morphometric analysis was performed with the CT analyzer.

### Histopathologic examination.

The mouse knees injected with P2C and P3C were performed for histopathologic examinations after μCT scanning. The routine fixation, decalcification, and paraffin embedding were performed. Tissue sections were stained with hematoxylin and eosin, and the joints were evaluated microscopically to assess synovial hypertrophy and cartilage and/or bone destruction in a blinded manner by two observers (Z.H. and T.J.) as described previously (54).

### Statistical analysis.

Student’s *t* test, one-way analysis of variance (ANOVA) with Tukey’s test, or Kruskal-Wallis test with Dunn’s multiple-comparison test was employed when appropriate to compare the differences between means. All the statistical analysis was performed with GraphPad Prism. The significant level was set as follows: a *P* value of >0.05 was considered not significant. In the figures, significant differences are indicated as described in the legends.

### Data availability.

The supporting data for this paper are openly available at figshare at https://figshare.com/articles/dataset/Bacterial_lipoproteins_shift_cellular_metabolism_to_glycolysis_in_macrophages_causing_bone_erosion/22140140 (upon publication).

## References

[1] Takeuchi O, Hoshino K, Kawai T, Sanjo H, Takada H, Ogawa T, Takeda K, Akira S. 1999. Differential roles of TLR2 and TLR4 in recognition of Gram-negative and Gram-positive bacterial cell wall components. Immunity 11:443–451. doi:10.1016/s1074-7613(00)80119-3. 10549626

[2] Nguyen MT, Götz F. 2016. Lipoproteins of Gram-positive bacteria: key players in the immune response and virulence. Microbiol Mol Biol Rev 80:891–903. doi:10.1128/MMBR.00028-16. 27512100 PMC4981669

[3] Nakayama H, Kurokawa K, Lee BL. 2012. Lipoproteins in bacteria: structures and biosynthetic pathways. FEBS J 279:4247–4268. doi:10.1111/febs.12041. 23094979

[4] Nguyen MT, Uebele J, Kumari N, Nakayama H, Peter L, Ticha O, Woischnig AK, Schmaler M, Khanna N, Dohmae N, Lee BL, Bekeredjian-Ding I, Götz F. 2017. Lipid moieties on lipoproteins of commensal and non-commensal staphylococci induce differential immune responses. Nat Commun 8:2246. doi:10.1038/s41467-017-02234-4. 29269769 PMC5740139

[5] Armbruster KM, Komazin G, Meredith TC. 2019. Copper-induced expression of a transmissible lipoprotein intramolecular transacylase alters lipoprotein acylation and the Toll-like receptor 2 response to Listeria monocytogenes. J Bacteriol 201:e00195-19. doi:10.1128/JB.00195-19. 30988036 PMC6560142

[6] Mohammad M, Nguyen MT, Engdahl C, Na M, Jarneborn A, Hu Z, Karlsson A, Pullerits R, Ali A, Götz F, Jin T. 2019. The YIN and YANG of lipoproteins in developing and preventing infectious arthritis by Staphylococcus aureus. PLoS Pathog 15:e1007877. doi:10.1371/journal.ppat.1007877. 31226163 PMC6608979

[7] Schultz M, Mohammad M, Nguyen MT, Hu Z, Jarneborn A, Wienken CM, Froning M, Pullerits R, Ali A, Hayen H, Götz F, Jin T. 2022. Lipoproteins cause bone resorption in a mouse model of Staphylococcus aureus septic arthritis. Front Microbiol 13:843799. doi:10.3389/fmicb.2022.843799. 35356518 PMC8959583

[8] Mohammad M, Na M, Hu Z, Nguyen MT, Kopparapu PK, Jarneborn A, Karlsson A, Ali A, Pullerits R, Götz F, Jin T. 2021. Staphylococcus aureus lipoproteins promote abscess formation in mice, shielding bacteria from immune killing. Commun Biol 4:432. doi:10.1038/s42003-021-01947-z. 33785850 PMC8010101

[9] O'Neill LA, Kishton RJ, Rathmell J. 2016. A guide to immunometabolism for immunologists. Nat Rev Immunol 16:553–565. doi:10.1038/nri.2016.70. 27396447 PMC5001910

[10] Escoll P, Buchrieser C. 2018. Metabolic reprogramming of host cells upon bacterial infection: why shift to a Warburg-like metabolism? FEBS J 285:2146–2160. doi:10.1111/febs.14446. 29603622

[11] Shi L, Salamon H, Eugenin EA, Pine R, Cooper A, Gennaro ML. 2015. Infection with Mycobacterium tuberculosis induces the Warburg effect in mouse lungs. Sci Rep 5:18176. doi:10.1038/srep18176. 26658723 PMC4674750

[12] Appelberg R, Moreira D, Barreira-Silva P, Borges M, Silva L, Dinis-Oliveira RJ, Resende M, Correia-Neves M, Jordan MB, Ferreira NC, Abrunhosa AJ, Silvestre R. 2015. The Warburg effect in mycobacterial granulomas is dependent on the recruitment and activation of macrophages by interferon-gamma. Immunology 145:498–507. doi:10.1111/imm.12464. 25807843 PMC4515130

[13] Wong Fok Lung T, Monk IR, Acker KP, Mu A, Wang N, Riquelme SA, Pires S, Noguera LP, Dach F, Gabryszewski SJ, Howden BP, Prince A. 2020. Staphylococcus aureus small colony variants impair host immunity by activating host cell glycolysis and inducing necroptosis. Nat Microbiol 5:141–153. doi:10.1038/s41564-019-0597-0. 31686028 PMC10184863

[14] Gillmaier N, Gotz A, Schulz A, Eisenreich W, Goebel W. 2012. Metabolic responses of primary and transformed cells to intracellular Listeria monocytogenes. PLoS One 7:e52378. doi:10.1371/journal.pone.0052378. 23285016 PMC3528701

[15] Escoll P, Song OR, Viana F, Steiner B, Lagache T, Olivo-Marin JC, Impens F, Brodin P, Hilbi H, Buchrieser C. 2017. Legionella pneumophila modulates mitochondrial dynamics to trigger metabolic repurposing of infected macrophages. Cell Host Microbe 22:302–316.e7. doi:10.1016/j.chom.2017.07.020. 28867389

[16] Czyż DM, Willett JW, Crosson S. 2017. Brucella abortus induces a Warburg shift in host metabolism that is linked to enhanced intracellular survival of the pathogen. J Bacteriol 199:e00227-17. doi:10.1128/JB.00227-17. 28559292 PMC5512224

[17] Siegl C, Prusty BK, Karunakaran K, Wischhusen J, Rudel T. 2014. Tumor suppressor p53 alters host cell metabolism to limit Chlamydia trachomatis infection. Cell Rep 9:918–929. doi:10.1016/j.celrep.2014.10.004. 25437549

[18] Rupp J, Gieffers J, Klinger M, van Zandbergen G, Wrase R, Maass M, Solbach W, Deiwick J, Hellwig-Burgel T. 2007. Chlamydia pneumoniae directly interferes with HIF-1alpha stabilization in human host cells. Cell Microbiol 9:2181–2191. doi:10.1111/j.1462-5822.2007.00948.x. 17490410

[19] Galli G, Saleh M. 2020. Immunometabolism of macrophages in bacterial infections. Front Cell Infect Microbiol 10:607650. doi:10.3389/fcimb.2020.607650. 33585278 PMC7879570

[20] Lachmandas E, Beigier-Bompadre M, Cheng SC, Kumar V, van Laarhoven A, Wang X, Ammerdorffer A, Boutens L, de Jong D, Kanneganti TD, Gresnigt MS, Ottenhoff TH, Joosten LA, Stienstra R, Wijmenga C, Kaufmann SH, van Crevel R, Netea MG. 2016. Rewiring cellular metabolism via the AKT/mTOR pathway contributes to host defence against Mycobacterium tuberculosis in human and murine cells. Eur J Immunol 46:2574–2586. doi:10.1002/eji.201546259. 27624090 PMC5129526

[21] Neustock P, Brand JM, Kruse A, Kirchner H. 1993. Cytokine production of the human monocytic cell line Mono Mac 6 in comparison to mature monocytes in peripheral blood mononuclear cells. Immunobiology 188:293–302. doi:10.1016/S0171-2985(11)80237-8. 8225390

[22] Stoll H, Dengjel J, Nerz C, Götz F. 2005. Staphylococcus aureus deficient in lipidation of prelipoproteins is attenuated in growth and immune activation. Infect Immun 73:2411–2423. doi:10.1128/IAI.73.4.2411-2423.2005. 15784587 PMC1087423

[23] Lewis AH, Bridges CS, Punia VS, Cooper AFJ, Puppi M, Lacorazza HD. 2021. Kruppel-like factor 4 promotes survival and expansion in acute myeloid leukemia cells. Oncotarget 12:255–267. doi:10.18632/oncotarget.27878. 33659038 PMC7899553

[24] Kibler E, Lavrinenko A, Kolesnik I, Stankevich K, Bolbasov E, Kudryavtseva V, Leonov A, Schepetkin I, Khlebnikov A, Quinn MT, Tverdokhlebov S. 2020. Electrosprayed poly(lactic-co-glycolic acid) particles as a promising drug delivery system for the novel JNK inhibitor IQ-1. Eur Polym J 127:109598. doi:10.1016/j.eurpolymj.2020.109598. 32372769 PMC7199471

[25] Anjum SA, Lawrence H, Holland JP, Kirby JA, Deehan DJ, Tyson-Capper AJ. 2016. Effect of cobalt-mediated Toll-like receptor 4 activation on inflammatory responses in endothelial cells. Oncotarget 7:76471–76478. doi:10.18632/oncotarget.13260. 27835611 PMC5363524

[26] Skabytska Y, Wolbing F, Gunther C, Koberle M, Kaesler S, Chen KM, Guenova E, Demircioglu D, Kempf WE, Volz T, Rammensee HG, Schaller M, Rocken M, Gotz F, Biedermann T. 2014. Cutaneous innate immune sensing of Toll-like receptor 2-6 ligands suppresses T cell immunity by inducing myeloid-derived suppressor cells. Immunity 41:762–775. doi:10.1016/j.immuni.2014.10.009. 25456159

[27] Gardiner J, Komazin G, Matsuo M, Cole K, Götz F, Meredith TC. 2020. Lipoprotein N-acylation in Staphylococcus aureus is catalyzed by a two-component acyl transferase system. mBio 11:e01619-20. doi:10.1128/mBio.01619-20. 32723923 PMC7387801

[28] Nguyen MT, Kraft B, Yu W, Demircioglu DD, Hertlein T, Burian M, Schmaler M, Boller K, Bekeredjian-Ding I, Ohlsen K, Schittek B, Götz F. 2015. The nSaα specific lipoprotein like cluster (lpl) of S. aureus USA300 contributes to immune stimulation and invasion in human cells. PLoS Pathog 11:e1004984. doi:10.1371/journal.ppat.1004984. 26083414 PMC4470592

[29] Nguyen MT, Schellerhoff LH, Niemann S, Schaumburg F, Herrmann M. 2022. Quiescence of human monocytes after affinity purification: a novel method apt for monocyte stimulation assays. Biomolecules 12:395. doi:10.3390/biom12030395. 35327587 PMC8945441

[30] Lachmandas E, Boutens L, Ratter JM, Hijmans A, Hooiveld GJ, Joosten LA, Rodenburg RJ, Fransen JA, Houtkooper RH, van Crevel R, Netea MG, Stienstra R. 2016. Microbial stimulation of different Toll-like receptor signalling pathways induces diverse metabolic programmes in human monocytes. Nat Microbiol 2:16246. doi:10.1038/nmicrobiol.2016.246. 27991883

[31] McGarry T, Biniecka M, Gao W, Cluxton D, Canavan M, Wade S, Wade S, Gallagher L, Orr C, Veale DJ, Fearon U. 2017. Resolution of TLR2-induced inflammation through manipulation of metabolic pathways in Rheumatoid Arthritis. Sci Rep 7:43165. doi:10.1038/srep43165. 28225071 PMC5320554

[32] Balic JJ, Albargy H, Luu K, Kirby FJ, Jayasekara WSN, Mansell F, Garama DJ, De Nardo D, Baschuk N, Louis C, Humphries F, Fitzgerald K, Latz E, Gough DJ, Mansell A. 2020. STAT3 serine phosphorylation is required for TLR4 metabolic reprogramming and IL-1beta expression. Nat Commun 11:3816. doi:10.1038/s41467-020-17669-5. 32732870 PMC7393113

[33] Kotake S, Sato K, Kim KJ, Takahashi N, Udagawa N, Nakamura I, Yamaguchi A, Kishimoto T, Suda T, Kashiwazaki S. 1996. Interleukin-6 and soluble interleukin-6 receptors in the synovial fluids from rheumatoid arthritis patients are responsible for osteoclast-like cell formation. J Bone Miner Res 11:88–95. doi:10.1002/jbmr.5650110113. 8770701

[34] Nishimoto N, Terao K, Mima T, Nakahara H, Takagi N, Kakehi T. 2008. Mechanisms and pathologic significances in increase in serum interleukin-6 (IL-6) and soluble IL-6 receptor after administration of an anti-IL-6 receptor antibody, tocilizumab, in patients with rheumatoid arthritis and Castleman disease. Blood 112:3959–3964. doi:10.1182/blood-2008-05-155846. 18784373

[35] Lam J, Takeshita S, Barker JE, Kanagawa O, Ross FP, Teitelbaum SL. 2000. TNF-alpha induces osteoclastogenesis by direct stimulation of macrophages exposed to permissive levels of RANK ligand. J Clin Invest 106:1481–1488. doi:10.1172/JCI11176. 11120755 PMC387259

[36] Monaco C, Nanchahal J, Taylor P, Feldmann M. 2015. Anti-TNF therapy: past, present and future. Int Immunol 27:55–62. doi:10.1093/intimm/dxu102. 25411043 PMC4279876

[37] Kelly B, O'Neill LA. 2015. Metabolic reprogramming in macrophages and dendritic cells in innate immunity. Cell Res 25:771–784. doi:10.1038/cr.2015.68. 26045163 PMC4493277

[38] Brook I, Reza MJ, Bricknell KS, Bluestone R, Finegold SM. 1978. Synovial fluid lactic acid. A diagnostic aid in septic arthritis. Arthritis Rheum 21:774–779. doi:10.1002/art.1780210706. 697948

[39] Kinugasa M, Kobayashi D, Satsuma S, Sakata R, Shinada Y, Kuroda R. 2020. The predictive value of synovial glucose level in septic arthritis. J Pediatr Orthop B 29:292–296. doi:10.1097/BPB.0000000000000628. 30882559

[40] Arnett TR. 2008. Extracellular pH regulates bone cell function. J Nutr 138:415S–418S. doi:10.1093/jn/138.2.415S. 18203913

[41] Meghji S, Morrison MS, Henderson B, Arnett TR. 2001. pH dependence of bone resorption: mouse calvarial osteoclasts are activated by acidosis. Am J Physiol Endocrinol Metab 280:E112–9. doi:10.1152/ajpendo.2001.280.1.E112. 11120665

[42] Teitelbaum SL. 2000. Bone resorption by osteoclasts. Science 289:1504–1508. doi:10.1126/science.289.5484.1504. 10968780

[43] Palsson-McDermott EM, Curtis AM, Goel G, Lauterbach MA, Sheedy FJ, Gleeson LE, van den Bosch MW, Quinn SR, Domingo-Fernandez R, Johnston DG, Jiang JK, Israelsen WJ, Keane J, Thomas C, Clish C, Vander Heiden M, Xavier RJ, O'Neill LA. 2015. Pyruvate kinase M2 regulates Hif-1alpha activity and IL-1beta induction and is a critical determinant of the Warburg effect in LPS-activated macrophages. Cell Metab 21:65–80. doi:10.1016/j.cmet.2014.12.005. 25565206 PMC5198835

[44] Krawczyk CM, Holowka T, Sun J, Blagih J, Amiel E, DeBerardinis RJ, Cross JR, Jung E, Thompson CB, Jones RG, Pearce EJ. 2010. Toll-like receptor-induced changes in glycolytic metabolism regulate dendritic cell activation. Blood 115:4742–4749. doi:10.1182/blood-2009-10-249540. 20351312 PMC2890190

[45] Aliprantis AO, Yang RB, Mark MR, Suggett S, Devaux B, Radolf JD, Klimpel GR, Godowski P, Zychlinsky A. 1999. Cell activation and apoptosis by bacterial lipoproteins through Toll-like receptor-2. Science 285:736–739. doi:10.1126/science.285.5428.736. 10426996

[46] Widaa A, Claro T, Foster TJ, O'Brien FJ, Kerrigan SW. 2012. Staphylococcus aureus protein A plays a critical role in mediating bone destruction and bone loss in osteomyelitis. PLoS One 7:e40586. doi:10.1371/journal.pone.0040586. 22792377 PMC3394727

[47] Cheng ML, Fong L. 2014. Effects of RANKL-targeted therapy in immunity and cancer. Front Oncol 3:329. doi:10.3389/fonc.2013.00329. 24432249 PMC3882875

[48] Huang R, Wang X, Zhou Y, Xiao Y. 2017. RANKL-induced M1 macrophages are involved in bone formation. Bone Res 5:17019. doi:10.1038/boneres.2017.19. 29263936 PMC5645773

[49] Moeller T, Wolfheimer S, Goretzki A, Scheurer S, Schulke S. 2019. NFkappaB- and MAP-kinase signaling contribute to the activation of murine myeloid dendritic cells by a flagellin A: allergen fusion protein. Cells 8:355. doi:10.3390/cells8040355. 30991709 PMC6523117

[50] Benito-Villalvilla C, Perez-Diego M, Angelina A, Kisand K, Rebane A, Subiza JL, Palomares O. 2022. Allergoid–mannan conjugates reprogram monocytes into tolerogenic dendritic cells via epigenetic and metabolic rewiring. J Allergy Clin Immunol 149:212–222.e9. doi:10.1016/j.jaci.2021.06.012. 34153371

[51] Otto AM. 2016. Warburg effect(s)—a biographical sketch of Otto Warburg and his impacts on tumor metabolism. Cancer Metab 4:5. doi:10.1186/s40170-016-0145-9. 26962452 PMC4784299

[52] Van Rooijen N, Sanders A. 1994. Liposome mediated depletion of macrophages: mechanism of action, preparation of liposomes and applications. J Immunol Methods 174:83–93. doi:10.1016/0022-1759(94)90012-4. 8083541

[53] Fatima F, Fei Y, Ali A, Mohammad M, Erlandsson MC, Bokarewa MI, Nawaz M, Valadi H, Na M, Jin T. 2017. Radiological features of experimental staphylococcal septic arthritis by micro computed tomography scan. PLoS One 12:e0171222. doi:10.1371/journal.pone.0171222. 28152087 PMC5289588

[54] Fei Y, Wang W, Kwiecinski J, Josefsson E, Pullerits R, Jonsson IM, Magnusson M, Jin T. 2011. The combination of a tumor necrosis factor inhibitor and antibiotic alleviates staphylococcal arthritis and sepsis in mice. J Infect Dis 204:348–357. doi:10.1093/infdis/jir266. 21742832

