# Supplementary material for: Bacterial Lipoproteins Shift Cellular Metabolism to Glycolysis in Macrophages Causing Bone Erosion
Source: Microbiol Spectr. 2023 May 16;11(3):e04293-22. doi: 10.1128/spectrum.04293-22 (PMC10269925; doi:10.1128/spectrum.04293-22)
Supplement: Supplemental file 1 — Fig. S1 to S6. Download spectrum.04293-22-s0001.pdf, PDF file, 0.2 MB [file spectrum.04293-22-s0001.pdf]

Supplementary data

Bacterial lipoproteins shift cellular metabolism to glycolysis in macrophages causing bone erosion

Minh-Thu Nguyen[1*,#], Zhicheng Hu[2,3*], Majd Mohammad[3], Hannah Schöttler[4], Silke Niemann[1], Michelle Schultz[3], Katarzyna Barczyk-Kahlert[5], Tao Jin[3], Heiko Hayen[4], Mathias Herrmann[1]

[1]Institute of Medical Microbiology, University Hospital Münster, Münster, Germany; [2]Center for Clinical Laboratories, The Affiliated Hospital of Guizhou Medical University, Guiyang, China; [3]Department of Rheumatology and Inflammation Research, Institute of Medicine, Sahlgrenska Academy, University of Gothenburg, Gothenburg, Sweden; [4]Institute of Inorganic and Analytical Chemistry, University of Münster, Münster, Germany. [5]Institute of Immunology, University of Münster, Münster, Germany.

Pam-Dhc-SK_4 (P1C) Pam$_2$CSK_4 (P2C) Pam$_3$CSK_4 (P3C) PamCys(Pam)SK_4 (Lyso)

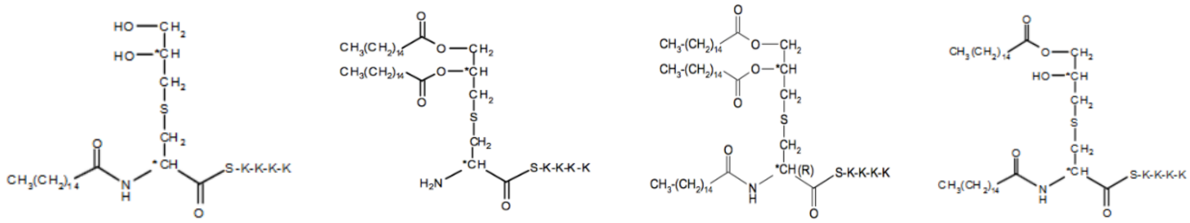

Supp. Figure S1. The biochemical structures of used lipopeptides as LPP analogues in this study. P1C (Pam-DHC-CSK4) is a synthetic monopalmitoylated lipopeptide. P2C (Pam$_2$CSK$_4$) is a synthetic dipalmitoylated lipopeptide that mimics the di-acylated amino terminus of LPP. P3C (Pam$_3$CSK$_4$) is a synthetic tripalmitoylated lipopeptide that mimics the tri-acylated amino terminus of LPP and Lyso (PamCysPamCSK$_4$), a synthetic monopalmitoylated N-palmitoylated lipopeptide mimics the Lyso-acylated amino terminus of LPP.

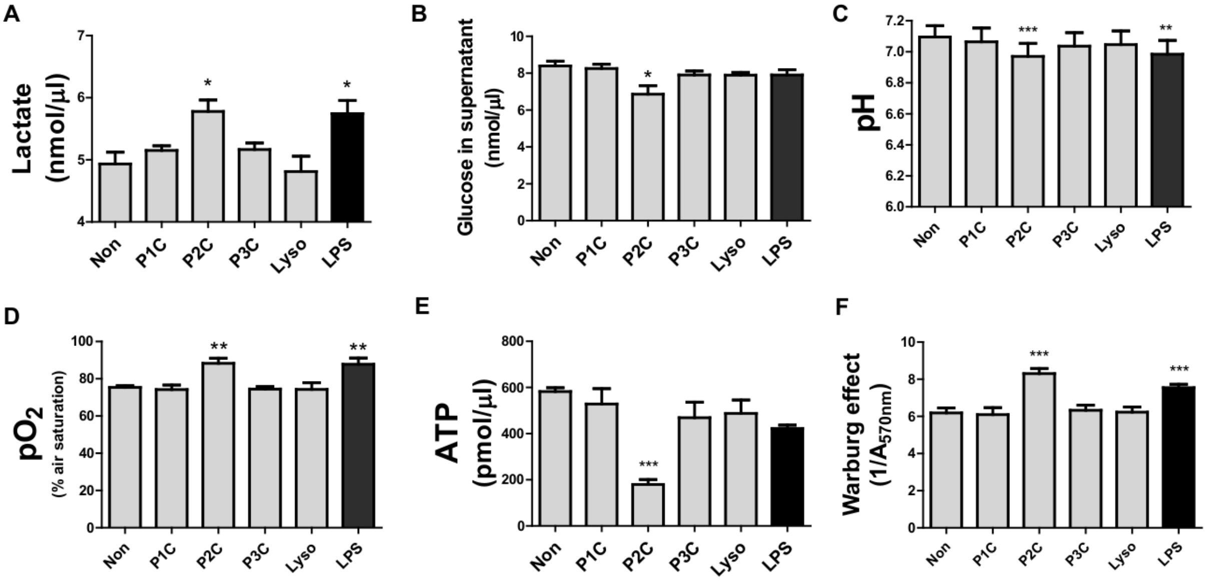

Supp. Figure S2. P2C confers significant effects on Lactate, glucose, pH, oxygen, ATP and the Warburg effect on human MM6 cells. Experimental conditions were as described in Methods, and the various assessments were performed in the supernatants of MM6 cells stimulated with 100 ng/mL of P1C, P2C, P3C, Lyso, or LPS, respectively, for either 24 h (**A**, **B, C, D**) or for 72 h (**E, F**) in RPMI medium. Control samples (non) were used without adding any stimulators. Three independent experiments were carried out in triplicated. Error bars represent SEM. Statistical significances were calculated between the treated cells compared to control (non) by one-way ANOVA: * $P< 0.05$, ** $P< 0.01$, *** $P< 0.001$.

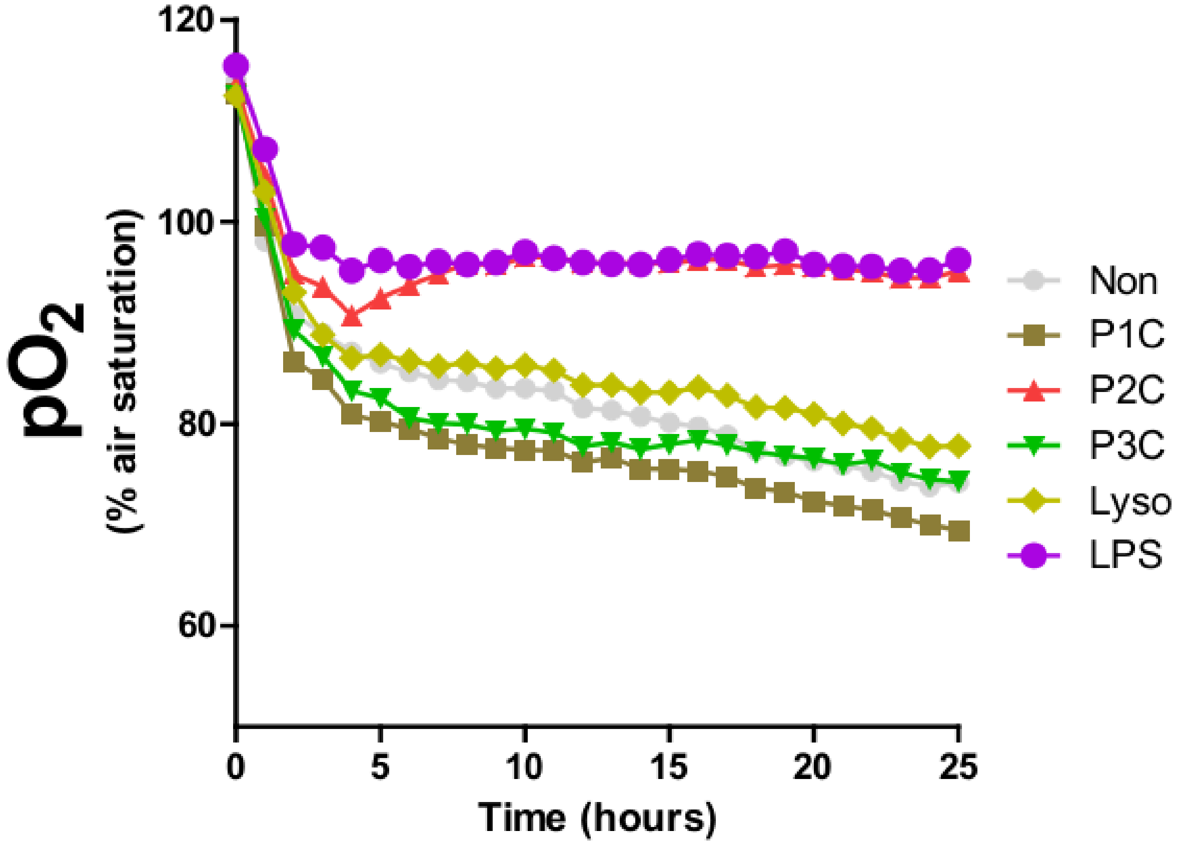

Supp. Fig. 3. Real-time oxygen concentration in MM6 cells untreated and treated with LPP analogues. 5x10^5 MM6 cells were incubated with 100 ng/ ml of LPP analogues, or LPS, in 1 ml of PRMI medium in 24 well plate Oxodish. The samples were measured hourly via SensorDish Reader (Presens).

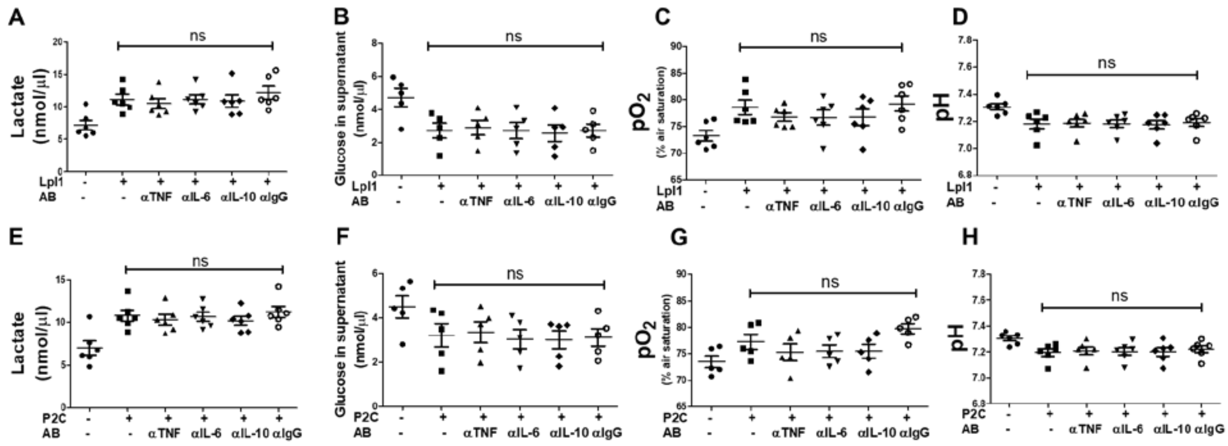

Supp. Fig. 4. Release Cytokines have no impact on the alteration of BMDM metabolism. 5x10^5 BMDM cells were incubated for 1 h with anti-mouse TNF, IL-6, and IL10 antibodies prior to stimulated with 300 ng/ml of Lpl1(+sp) (**A-D**) or with 100 ng/ml of P2C (**E-F**) for 24 h. The data was obtained from cells isolated from 5-6 mice. Error

bars indicate mean ± SEM. Statistical significances were calculated between the treated cells by one-way ANOVA using Kruskal- Wallis Comparison Test; ns, presented for not significant, P> 0.05.

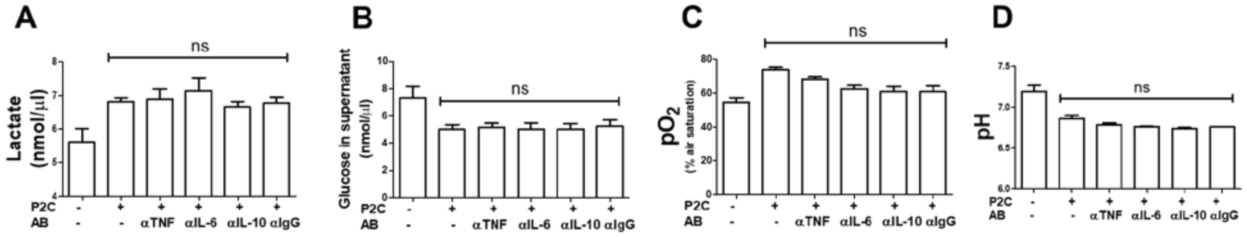

Supp. Fig. 5. Release Cytokines have no impact on the alteration of MM6 metabolism. $5x10^5$ MM6 cells were incubated for 1 h with anti-human TNF, IL-6, and IL10 antibodies prior to stimulated with 100 ng/ml of P2C for 24 h. Three independent experiments were carried out. Error bars represent SEM. Statistical significances were calculated between the treated cells by one-way ANOVA using Tukey's Multiple Comparison Test; ns, presented for not significant, P> 0.05.

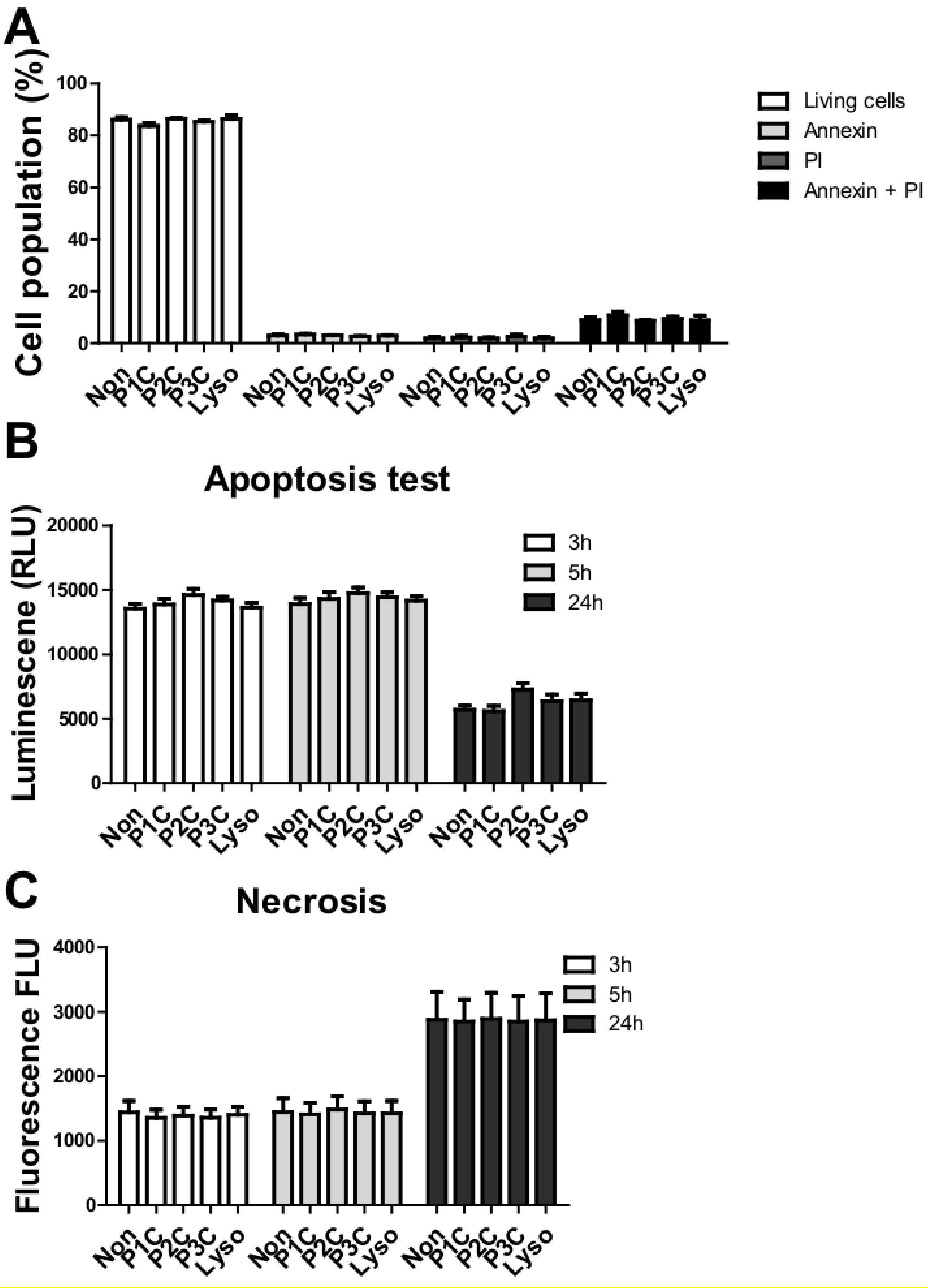

Supp. Fig 6. LPP analogues have no effect on apoptosis and necrosis in MM6. **(A)** MM6 cells populations of living cell, apoptotic cells (stained with Annexin V), necrotic cells (stained with PI), and apoptotic/necrotic cells (stained with Annexin V and PI) were measured by flow cytometry. MM6 cells were treated with 100 ng/ ml of

LPP analogues for 24 h. The proportion of apoptotic (**B**) and necrotic (**C**) cells was further determined by RealTime-Glo Annexin V Promega kit. LPP analogue-treated MM6 cells were measured after 3, 5 and 24 h stimulation. The control samples (non) are untreated cells. Three independently experiments were carried out in duplicated. Error bars represent SEM. Statistical significances were calculated between the treated cells compared to control (non) by one-way ANOVA. No significant differences between the various determinations was observed.
